# Patterns of Circulating piRNAs in the Context of a Single Bout of Exercise: Potential Biomarkers of Exercise-Induced Adaptation?

**DOI:** 10.3390/ncrna11030046

**Published:** 2025-06-16

**Authors:** Caroline Eva Riedel, Javier Ibáñez, Annunziata Fragasso, Angelika Schmitt, Manuel Widmann, Felipe Mattioni Maturana, Andreas M. Niess, Barbara Munz

**Affiliations:** 1 Department of Sports Medicine, Medical Clinic, University Hospital Tübingen, Hoppe-Seyler- Straße. 6, D-72076 Tübingen, Germanyannunziata.fragasso@med.uni-tuebingen.de (A.F.); manuel.widmann@med.uni-tuebingen.de (M.W.); felipe.mattioni@med.uni-tuebingen.de (F.M.M.); andreas.niess@med.uni-tuebingen.de (A.M.N.); 2 Interfaculty Research Institute for Sport and Physical Activity, Eberhard Karls University of Tübingen, D-72074 Tübingen, Germany

**Keywords:** training adaptation, piRNA, epigenetics, endurance training, exercise biomarkers

## Abstract

Background: Physical activity induces a range of physiological and molecular adaptations, particularly affecting skeletal muscle and the cardiovascular system, regulating both tissue architecture and metabolic pathways. Emerging evidence suggests that PIWI-interacting RNAs (piRNAs) may serve as potential biomarkers for these adaptations. Here, we analyzed piRNA patterns in the context of exercise. Methods: This study selected eight participants of the iReAct study (DRKS00017446) for piRNA analysis. Baseline assessments included demographic profiling and fitness evaluation, particularly maximal oxygen uptake (V̇O2max) assessment. In addition, blood samples were collected pre- and (for six of the eight participants) post- standard reference training sessions. Subsequently, subjects underwent 6-week training protocols, employing standardized high-intensity interval training (HIIT) and moderate-intensity continuous training (MICT) regimens. Next, RNA sequencing was conducted to identify differentially expressed piRNAs, and correlation analyses were performed between piRNA expression patterns and training-associated changes in V̇O2max. Finally, to identify piRNAs potentially of interest in the context of exercise, different screening procedures were applied. Results: There were unique and specific changes in individual piRNA expression levels in response to exercise. In addition, we could define correlations of piRNA expression patterns, namely of piR-32886, piR-33151, piR-12547, and piR-33074, with changes in V̇O2max. These correlations did not reach significance in the small sample size of this pilot study, but might be verified in larger, confirming studies. Conclusions: This hypothesis-generating study identifies characteristic piRNA patterns in the context of exercise. Their significance as biomarkers is yet to be determined.

## 1. Introduction

Physical activity promotes health and reduces the risk of chronic diseases by inducing numerous adaptations in the body. These adaptations primarily occur in skeletal muscle and the cardiovascular system, particularly with regard to tissue architecture and energy metabolism, enhancing both physical and mental health [1,2,3,4,5]. Currently, the World Health Organization recommends approximately 150 min of moderate exercise per week for pregnant and postpartum women, 150–300 min per week for adults and older adults, and around 60 min per day for children and adolescents [6].

When physical activity is carried out systematically, planned and target-oriented, it is generally referred to as training [7,8]. Training effects depend significantly on exercise modality, duration, and intensity. For example, endurance training can be carried out as high-intensity interval training (HIIT), alternating between periods of high-effort exercise and recovery, offering cardiovascular and metabolic benefits [9,10], or moderate-intensity continuous training (MICT), consisting of prolonged, steady-state exercise at moderate intensity. Both endurance training modalities influence metabolic thresholds, namely lactate thresholds #1 (the first detectable rise in lactate levels) and #2 (the point where lactate levels rise rapidly, since lactate production exceeds the body’s clearance capacity), which are indicators of the body’s energy utilization during exercise. Metabolic adaptation to training involves various processes, such as lactate clearance and mitochondrial function [10,11,12,13].

These physiological changes are based on molecular adaptation reactions [14], particularly modifications in the activity of signal transduction pathways, which lead to altered expression patterns of genes encoding both structural proteins and (metabolic) enzymes, namely increases or decreases in expression of specific genes or expression of different isoforms. These processes can be regulated by epigenetic mechanisms [1,15]. The term ‘epigenetics’ refers to heritable but reversible modifications in gene expression that do not alter the DNA sequence [16]. Among the key epigenetic players, small non-coding RNAs (sncRNAs), like microRNAs (miRNAs) and PIWI-interacting RNAs (piRNAs), are emerging as critical regulators of gene expression through post-transcriptional silencing mechanisms [17]. While miRNAs have been widely studied in the context of exercise [14,18,19,20,21], piRNAs remain underexplored [14].

piRNAs were first characterized in germline cells of fruit flies, where they regulate transposable elements and contribute to genome stability [17,22,23]. PIWI-interacting RNAs (piRNAs) are small, single-stranded non-coding RNAs that are 24–35 nucleotides in length. piRNAs can be categorized as sense or antisense based on their strand orientation. Sense piRNAs often have an adenine residue at the tenth nucleotide, while antisense piRNAs typically feature uridine at their 5′ ends. These distinct nucleotide preferences are essential for their biogenesis and function within the PIWI protein complex [17,22,24,25]. Recent research indicates that piRNAs and PIWI proteins, traditionally associated with germline maintenance, also play significant roles in somatic cells, as they influence mRNA stability, translation, and retrotransposon silencing in various tissues [26]. Additionally, studies have shown that the piRNA pathway responds to environmental signals, establishing intergenerational responses to environmental conditions. This indicates that piRNAs may participate in epigenetic modulation during stress responses, including those induced by physical activity [27]. Furthermore, evidence from studies in several model organisms suggests that piRNAs might potentially influence transcriptional activity and chromatin modifications in response to environmental stimuli, such as physical exercise [28,29].

In this study, we investigated piRNA expression patterns as potential biomarkers for adaptation reactions in response to physical exercise, correlating these patterns with physiological adaptations to endurance training, particularly changes in maximal oxygen uptake (V̇O2max).

## 2. Results

### 2.1. Differentially Expressed piRNAs

**Figure 1:**
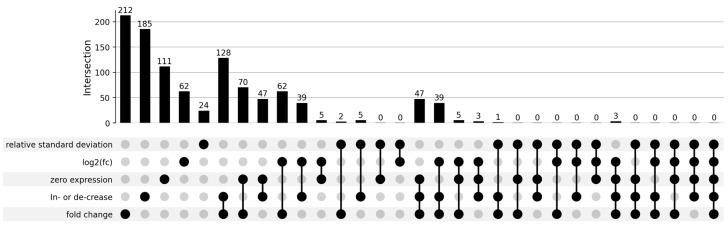
Differentially expressed piRNAs filtered by five distinct criteria. The number of piRNAs identified by five selection criteria: ‘relative standard deviation’, ‘log2(fc)’, ‘zero expression’, ‘increase/decrease’, and ‘fold change’. The bar graph indicates the number of piRNAs meeting each individual criterion and the different criteria combinations (labelled in black). The ‘fold change’ criterion identified the highest number of piRNAs (212), with significant overlap observed with the ‘increase/decrease’ (128) and ‘zero expression’ (70) criteria. The ‘relative standard deviation’ criterion showed minimal overlap with others, identifying 24 piRNAs. Only three piRNAs satisfied four criteria (‘log2(fc)’, ‘fold change’, ‘zero expression’, and ‘increase/decrease’). An upset plot was used for visualizing these data, highlighting the degree of complexity and distinctiveness of each criterion.

**Figure 2:**
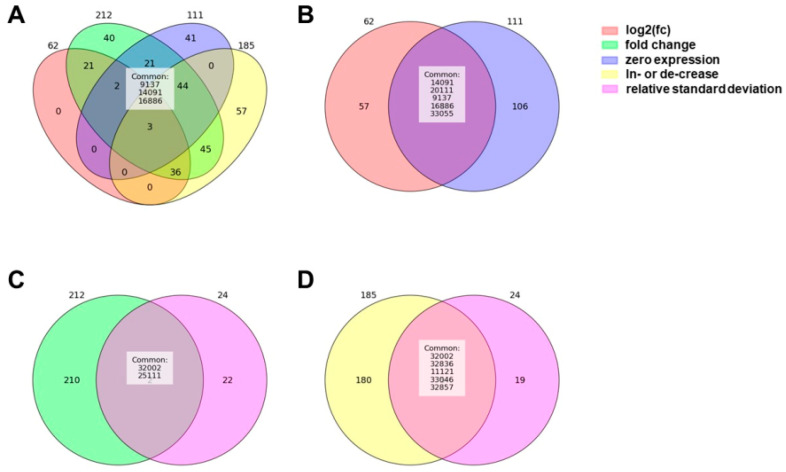
Venn diagrams illustrating the relationships between the five selection criteria—‘log2(fc)’, ‘fold change’, ‘zero expression’, ‘increase/decrease’, and ‘relative standard deviation’. (**A**) Three piRNAs (hsa-piR-9137, hsa-piR-14091, hsa-piR-16886) satisfied the ‘log2(fc)’, ‘fold change’, ‘zero expression’, and ‘increase/decrease’ criteria. (**B**) Five piRNAs (hsa-piR-20111, hsa-piR-33055, hsa-piR-14091, hsa-piR-9137, hsa-piR-16886) met both the ‘log2(fc)’ and ‘zero expression’ criteria. (**C**) Minimal overlap was observed between the ‘fold change’ and ‘relative standard deviation’ criteria, with only two piRNAs (hsa-piR-25111, hsa-piR-32002) identified. (**D**) Five piRNAs (hsa-piR-32857, hsa-piR-32836, hsa-piR-32002, hsa-piR-11121, hsa-piR-33046) met both the ‘relative standard deviation’ and ‘increase/decrease’ criteria. Numbers indicate total piRNAs selected for each criterion, highlighting overlaps across the different requirements.

An overview of the piRNAs meeting the five different criteria is shown in Figure 1 and Figure 2 and in Tables S1 and S2.

No piRNA species met all five criteria. Three piRNAs (hsa-piR-9137, hsa-piR-14091, hsa-piR-16886) satisfied the ‘log2(fc)’, ‘fold change’, ‘zero expression’, and ‘increase/decrease’ criteria (Figure 2A). The ‘relative standard deviation’ requirement showed the least overlap with all other criteria. The ‘fold change’ requirement included the most piRNA species and exhibited the highest overlap with all other criteria, except for the ‘relative standard deviation’ requirement.

Applying the ‘log2(fc)’ requirement resulted in 62 piRNAs. Since the ‘fold change’ requirement is a less stringent version of the ‘log2(fc)’ requirement, the list of piRNAs under the ‘fold change’ requirement represents a subset of the piRNAs identified by the ‘log2(fc)’ requirement. No overlap was observed between the piRNAs identified by the ‘log2(fc)’ requirement and those identified by the ‘relative standard deviation’ requirement. Only five piRNAs from the ‘log2(fc)’ list were also found in the ‘zero expression’ list (hsa-piR-20111, hsa-piR-33055, hsa-piR-14091, hsa-piR-9137, hsa-piR-16886) (Figure 2B). Of the 62 piRNAs identified by the ‘log2(fc)’ requirement, 39 were also found in the ‘increase/decrease’ list. This overlap represents 63% of the piRNAs meeting the ‘log2(fc)’ requirement and also the ‘increase/decrease’ requirement. Approximately half of the 39 piRNAs that met both criteria displayed expression values close to or equal to zero across all baseline samples.

As mentioned earlier, piRNA species meeting the ‘log2(fc)’ requirement are fully included in the 212 piRNAs identified by the ‘fold change’ requirement. These 212 piRNAs represent more than half of the 411 piRNAs found in total. Expression data for these 212 piRNAs indicate that many of their expression values were also close to or equal to zero, similar to those for the piRNAs identified by the ‘log2(fc)’ requirement. Concordance of the ‘fold change’ requirement with the ‘relative standard deviation’ requirement was minimal, with only two piRNAs (hsa-piR-25111, hsa-piR-32002) identified (Figure 2C). By contrast, overlap with the ‘zero expression’ requirement was much greater than with the ‘log2(fc)’ requirement. A total of 70 shared piRNAs were identified, representing approximately 33% of the piRNAs identified by the ‘fold change’ requirement and around 63% of the piRNAs identified by the ‘zero expression’ requirement. All 70 of these piRNAs showed expression values equal to or near zero across all baseline samples.

Furthermore, 128 individual piRNAs identified by the ‘fold change’ requirement also satisfied the ‘increase/decrease’ requirement. This represents more than half of the piRNAs from the ‘fold change’ list (60%) and about 69% of the piRNAs from the ‘increase/decrease’ list. More than half of these 128 piRNAs displayed expression values close to or equal to zero across all baseline samples.

The ‘relative standard deviation’ requirement, as mentioned earlier, showed the least overlap with all other criteria. Only 24 piRNAs met this criterion. Additionally, only five of these piRNAs (hsa-piR-32857, hsa-piR-32836, hsa-piR-32002, hsa-piR-11121, hsa-piR-33046) were found to overlap with the ‘increase/decrease’ requirement (Figure 2D).

Applying the ‘zero expression’ requirement led to the identification of 111 piRNA species, approximately half the number found with the ‘fold change’ requirement. The ‘zero expression’ requirement showed extensive overlap with the ‘increase/decrease’ requirement, with 42% (47) of the piRNAs from the ‘zero expression’ list also satisfying the ‘increase/decrease’ requirement. No overlap was observed between the ‘zero expression’ and ‘relative standard deviation’ criteria. Expression values for these piRNAs were all zero or near zero across all baseline samples.

Finally, the ‘increase/decrease’ requirement identified 185 individual piRNAs, a number comparable to the 212 piRNAs identified by the ‘fold change’ requirement.

### 2.2. Plot-Based piRNA Selection

Nine of the twenty-four piRNA species that satisfied the ‘relative standard deviation’ criterion were selected for detailed analysis. These piRNAs demonstrated consistent expression patterns across all subjects for the three sample time points: ‘at rest’, ‘immediately’, and ‘+3 h’. The identified piRNAs were hsa-piR-1677, hsa-piR-11119, hsa-piR-25111, hsa-piR-32835, hsa-piR-32874, hsa-piR-32956, hsa-piR-33036, hsa-piR-33041, and hsa-piR-33074. Three additional piRNAs identified using the ‘relative standard deviation’ criterion exhibited homogeneous expression patterns for most subjects, but significant expression variability was observed in at least two individuals. Consequently, these piRNAs—hsa-piR-32002, hsa-piR-32836, and hsa-piR-33013—were excluded from further detailed analysis.

Among the piRNA species identified by the ‘log2(fc)’ criterion, only 4 out of 62 displayed uniform expression values and were selected for further investigation. These piRNAs were hsa-piR-12547, hsa-piR-32886, hsa-piR-32941, and hsa-piR-33151. However, eleven additional piRNAs from the ‘log2(fc)’ group showed homogeneous expression patterns in most subjects, but failed to meet all inclusion criteria. As a result, these piRNAs—hsa-piR-1056, hsa-piR-1496, hsa-piR-12319, hsa-piR-33055, hsa-piR-33048, hsa-piR-32180, hsa-piR-28258, hsa-piR-28085, hsa-piR-27080, hsa-piR-20101, and hsa-piR-12390—were excluded from further characterization.

Detailed data on the thirteen individual piRNAs finally selected for more extensive analysis are summarized in Table S3.

**Figure 3:**
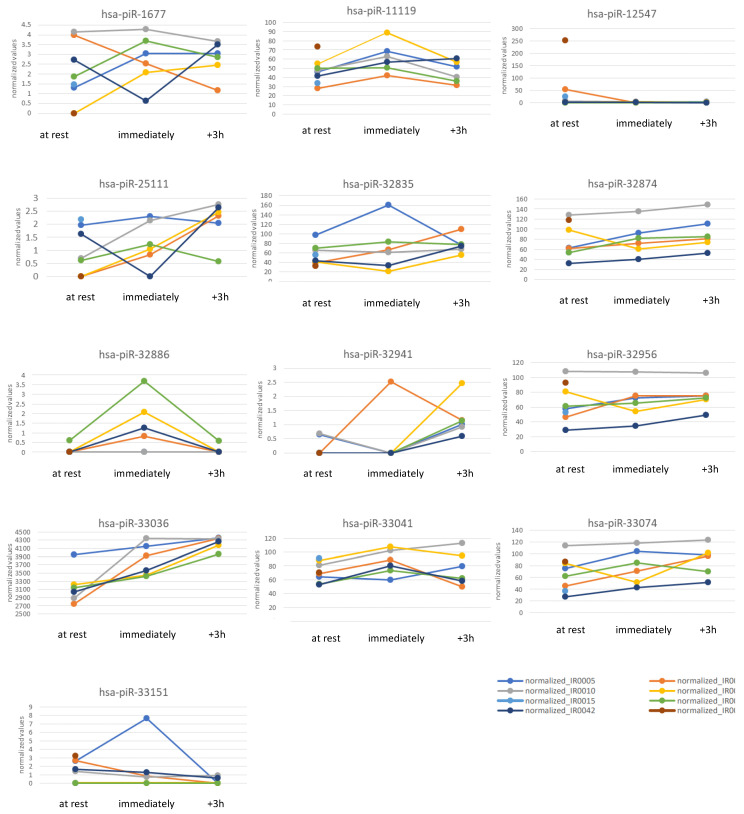
Kinetics of normalized expression of the *n* = 13 selected piRNAs. (‘at rest’: *n* = 8; ‘immediately’ and ‘+3 h’: *n* = 6).

Most selected piRNAs demonstrated increasing expression levels during exercise. Notably, expression of hsa-piR-11119 and hsa-piR-32886 quite consistently and sharply increased immediately after exercise—to then decrease again, with hsa-piR-32886 showing expression values ‘at rest’ and ‘+3 h’ that were often close to zero, though this piRNA was still included in the analysis due to its consistent expression pattern. Hsa-piR-32941, hsa-piR-33074, and hsa-piR-33036 all displayed clear and steady increases in expression from ‘at rest’ to 3 h post-exercise, with no decreases observed across any of the participants. Hsa-piR-33036 exhibited particularly high expression values (all above 2500) and a low relative standard deviation at the 3-hour post-exercise time point, with expression values clearly increasing and converging across all participants. A similar expression pattern was observed for hsa-piR-25111, which, according to piRNAdb, has previously been detected in tumor tissue. However, here, induction was only short-lived and moderate for two participants. By contrast, hsa-piR-33151 and hsa-piR-12547 were the only piRNAs to show a general decrease in expression in response to exercise. Table S3 and Figure 3 illustrate the expression kinetics of the selected piRNAs.

### 2.3. piRNA Characterization

A search of the piRNAdb database identified matches in various tissues, including testis, lung, and liver [30]. Seven of the piRNAs identified in the study by Mei et al. (2015) [31] were found in the Data S3 of their paper as ‘novel piRNA-like small RNAs’. All selected piRNAs exhibited the characteristic piRNA length. Notably, four piRNAs displayed the characteristic 5′ uridine typical of antisense piRNAs (hsa-piR-11119, hsa-piR-25111, hsa-piR-33036, hsa-piR-33041), while three piRNAs showed an adenine at their 10th nucleotide position, indicative of sense piRNAs (hsa-piR-1677, hsa-piR-11119, hsa-piR-25111). Additionally, four piRNAs (hsa-piR-32835, hsa-piR-32874, hsa-piR-32941, hsa-piR-32956) contained adenine near the 10th nucleotide (at the 9th or 11th position).

A literature search of the piRNAdb database identified three main studies referencing the piRNAs selected in the earlier steps. The first study, “A germline-specific class of small RNAs binds mammalian Piwi proteins” by Girard et al. (2006) [32], mentions hsa-piR-1677, hsa-piR-11119, hsa-piR-12547, and hsa-piR-25111. The second study, “A piRNA-like small RNA interacts with and modulates p-ERM proteins in human somatic cells” by Mei et al. (2015) [31], already mentioned above, includes hsa-piR-32835, hsa-piR-32874, hsa-piR-32956, hsa-piR-33036, hsa-piR-33041, hsa-piR-32941, and hsa-piR-32886. The third study, “Specific patterns of PIWI-interacting small noncoding RNA expression in dysplastic liver nodules and hepatocellular carcinoma” by Rizzo et al. (2016) [33], references hsa-piR-33074 and hsa-piR-33151.

### 2.4. Correlation with ΔV̇O2max

**Figure 4:**
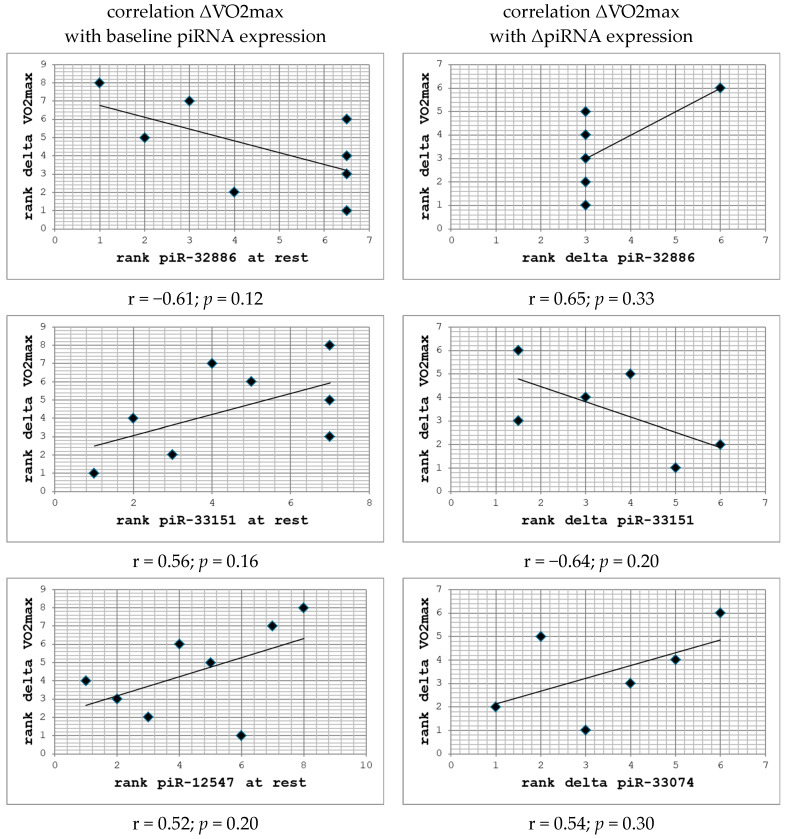
Correlation of piRNA patterns with ΔV̇O2max between baseline and FU1. Diagrams illustrate correlations with ‘at rest’ expression (*n* = 8) and with changes between ‘at rest’ and ‘+3 h’ (*n* = 6) for which correlation coefficients (Spearman) of either >0.5 or <−0.5 were found.

Probably due to the small sample size, there were no significant correlations between piRNA expression patterns and training-associated changes in ΔV̇O2max. However, we observed some interesting trends that might be verified in larger, confirmatory analyses: When correlation was carried out with baseline piRNA expression data, only three of the selected piRNA species had a correlation coefficient of r ≥ |0.5|: hsa-piR-32886 (r = −0.6088), hsa-piR-33151 (r = 0.56114), and hsa-piR-12547 (r = 0.5238). Among these, hsa-piR-32886 had the highest degree of correlation; however, the functional significance of this finding is probably limited, since 4/8 subjects displayed zero expression at baseline (Figure 4). Similarly, when examining correlations between changes in piRNA expression values and ΔV̇O2max, only three piRNAs had r ≥ |0.5|: hsa-piR-33151 (r = −0.6377), hsa-piR-32886 (r = 0.6546), and hsa-piR-33074 (r = 0.5429) (Figure 4). Again, in the case of hsa-piR-32886, it should be emphasized that for all four subjects that displayed a sharp peak immediately after exercise, expression levels of this piRNA had already returned to baseline at time point ‘+3 h’, limiting the significance of correlation with deltas between ‘at rest’ and ‘+3 h’ (Table S3).

## 3. Discussion

In this pilot study, we have established and evaluated selection procedures for the analysis of individual piRNA species as biomarkers of exercise-induced adaptation.

For this purpose, we employed blood samples of *n* = 6–8 female subjects, who had performed a single bout of exercise on a bike ergometer. Samples were taken pre- and immediately post-exercise, as well as 3 h later. Using small RNA sequencing, we could identify *n* = 411 different piRNA species as being expressed in these samples. Our first goal was then to filter this dataset in order to identify piRNA species differentially expressed in the context of exercise, since these might be functionally linked to the process of exercise adaptation. For this purpose, we applied different selection criteria and eventually defined *n* = 13 piRNA species for further evaluation in future, confirmatory analyses, employing larger cohorts and alternative methods for piRNA detection, such as qPCR. Our second goal was to identify piRNA species whose expression correlated with training adaptation. For this purpose, following initial testing in the context of an acute bout of exercise as described above, subjects underwent a six-week endurance training program on a bike ergometer, and their gains in V̇O2max were correlated with piRNA patterns in the context of acute exercise, as described above. Though not reaching significance, we were able to define four individual piRNA species, whose expression, either at baseline and/or with regard to changes in the context of acute exercise, showed interesting correlations with ΔV̇O2max, rendering them promising candidates for biomarkers in the context of individual exercise adaptation. Despite the fact that these results are preliminary, they indicate that piRNAs might be functionally relevant and also serve as potential biomarkers in the context of exercise adaptation.

Initially, we applied five pre-selection criteria to identify piRNA species potentially relevant to training adaptation, each with distinct advantages and limitations. No piRNA met all five criteria, and only three (hsa-piR-9137, hsa-piR-14091, hsa-piR-16886) fulfilled four of them, (‘log2(fc)’, ‘fold change’, ‘zero expression’ and‚‘increase/decrease’), marking these as potentially of interest, while also highlighting the potential value of piRNAs meeting two or three criteria.

The ‘log2(fc)’ requirement is a commonly used method to identify differentially expressed piRNAs. Interestingly, there is no standard consensus on the criteria for selecting ranges, leaving authors to define them subjectively, i.e., based on their study goals. Here, log2(fc) identified piRNAs with expression changes exceeding fourfold. However, high fold changes often result from ratios of near-zero values, raising concerns about measurement artifacts.

The related ‘fold change’ requirement, with a less stringent threshold (+/−2), applied to a larger number of piRNAs, nevertheless also included many with low or zero expression, limiting its utility for identifying biologically relevant piRNAs.

While specific studies directly applying the relative standard deviation requirement to piRNA selection are scarce, some have underscored the relevance of using statistical parameters like relative standard deviation to evaluate expression variability [34]. In this study, the relative standard deviation requirement aimed to find piRNAs with consistent expression patterns across all participants. Unlike absolute standard deviation, it standardizes deviations relative to mean expression values, offering a uniform cutoff. Few piRNAs met this requirement, suggesting uniform expression patterns are rather rare. Interestingly, piRNAs identified by this requirement rarely overlapped with those meeting stricter fold-change criteria, implying distinct biological characteristics.

Excluding piRNA species with expression values close to or equal to zero is a common practice to eliminate potential noise [35]. Here, the ‘zero expression’ requirement targeted piRNAs not expressed before or at one of the two time points after exercise. However, most identified piRNAs had zero or near-zero expression across all samples, rendering this requirement less informative. Similarly, the increase/decrease requirement, which identified piRNAs with increasing or decreasing average expression values, included numerous piRNAs with low or zero expression, raising doubts about their biological significance.

Overlaps between criteria varied, with the ‘fold change’ and ‘increase/decrease’ criteria identifying the most piRNA species. However, again, many of these piRNAs had low expression levels, reducing their potential biological relevance. By contrast, the combination of ‘log2(fc)’ and ‘relative standard deviation’ appeared most promising for identifying biologically meaningful piRNAs.

In addition, some piRNA species met both the ‘fold change’ and the ‘zero expression’ criteria. These are the piRNAs that showed no expression at one of the time points, but a significant ‘fold change’ between two of the others. In fact, these piRNAs might be specifically interesting for consideration as biomarkers in the future, since their expression (‘yes/no’) at certain time points might be an ‘easy-to-assess’ predictor of particular adaptation reactions.

Overall, these findings underscore the importance of combining complementary criteria to balance sensitivity and specificity, ensuring the identification of piRNAs with both significant expression changes and consistent expression patterns across samples.

Next, line graphs were used to visualize individual piRNA expression patterns for each participant. piRNAs without clear trends, determined through visual inspection, were excluded due to the inability to draw conclusions. However, it is obvious that the limited sample size (six to eight participants, with two providing data only at baseline) introduced a high degree of variability and restricted the robustness of these conclusions.

To improve objectivity, ‘uniform expression’ was defined as a consistent trend in all or all but one participant. This approach is commonly employed in gene expression studies to pinpoint genes with stable expression, which can serve as reliable reference points or biomarkers [36]. However, this strict requirement risked excluding potentially relevant piRNAs. As a compromise, piRNAs with no expression in up to two participants, but consistent trends in others, were also included. Expression values near zero were excluded to minimize the influence of noise. Discrepancies in trends when considering different time points (‘at rest’, ‘immediately’, and ‘+3 h’) further complicated the selection process, necessitating the use of multiple plots for decision-making. Obviously, despite careful inspection of the plots, aiming at reducing bias, due to the lack of a clear definition of ‘trend’, this approach contained a subjective element and was thus not entirely objective. It was chosen against the background of the pilot, hypothesis-generating character of our study, and the low number of subjects, particularly to minimize the chances of excluding potentially relevant piRNAs that might be interesting for further testing in larger, confirmatory analyses.

Ultimately, 9 out of the 24 piRNA species meeting the ‘relative standard deviation’ requirement were selected for further characterization, as this requirement identified piRNAs with consistent expression trends across participants. Notably, however, three of the excluded piRNAs (hsa-piR-32003, hsa-piR-32836, hsa-piR-33013) showed mostly homogeneous patterns, and might warrant further investigation in future studies.

Remarkably, several of the piRNAs that had been identified by the ‘relative standard deviation’ criterion displayed relatively high standard deviations at one or two of the other time points. This was probably a result of the small sample size and needs reassessment in future, larger studies. In addition, only four piRNAs meeting the ‘log2(fc)’ requirement were selected, as many had expression values near zero, which limited their interpretability. Despite this, several piRNAs displayed relatively homogeneous patterns without meeting the strict uniformity requirement, suggesting potential relevance for future research.

In summary, while the selection criteria applied were stringent and may have excluded some interesting piRNAs, this approach was necessary to focus the analysis. As a hypothesis-generating study, the primary aim was to define and evaluate selection criteria, laying the groundwork for a more comprehensive and refined search strategy in future studies.

The sequencing data used in this study were based on piRNAdb, an NCBI-associated piRNA database. NCBI provides vast molecular biology, biochemistry, and genetics resources to the scientific community. Other databases, like piRBase, refer to the same piRNAs, but use different naming conventions due to their independent sources. Evidence from prior studies, such as those by Yang et al. (2015) [37] and Freedman et al. (2016) [38], supports the notion that the small RNA fragments identified via next-generation sequencing (NGS) in this study are indeed piRNAs. These findings suggest the potential of piRNAs as blood-based biomarkers for training-induced adaptation mechanisms, a promising area for further research.

While little is known about piRNAs in the context of training, recent studies highlight their possible functional roles in this context. For example, piRNAs may regulate heart regeneration and hypertrophy, making them potential biomarkers for pathological cardiac hypertrophy [39,40]. Furthermore, their expression in skeletal muscle tissue underscores their relevance in sports and exercise contexts [40].

Taken together, the analysis conducted here shows that piRNAs can be assessed from human blood of healthy subjects and exhibit exercise-responsive changes. However, further studies are necessary to confirm these findings, ideally with larger sample sizes and alternative methods such as qPCR. In addition, it would be interesting to test whether piRNAs not only respond to exercise, but also to other physiological challenges, such as stress or metabolic alterations. Concerns regarding the authenticity of piRNAs detected in blood plasma via sequencing [41] were addressed by analyzing piRNA characteristics. Three selected piRNAs in this study (hsa-piR-1677, hsa-piR-11119, and hsa-piR-12547) were listed as ambiguous by Tosar et al. (2018) [41], suggesting they may not be genuine piRNAs. However, all three exhibit piRNA-characteristic lengths, and hsa-piR-11119 additionally shows a 5′ uridine and 10th nucleotide adenine bias. Hsa-piR-1677 displays adenine bias at the 10th position, suggesting it is a sense piRNA, whereas hsa-piR-12547 only exhibits the characteristic length. Nevertheless, without evidence of interaction with PIWI proteins, the classification of these RNAs remains uncertain.

By contrast, ten piRNAs not listed as ambiguous in Tosar et al. (2018) [41] likely represent authentic piRNAs. Notably, hsa-piR-33036 and hsa-piR-33041 exhibit characteristic 5′ uridine or 10th nucleotide adenine biases, with hsa-piR-33036 also displaying high expression values and uniform response to exercise. Similar conclusions apply to hsa-piR-32941, hsa-piR-33074, and hsa-piR-33151, all of which showed consistent expression trends with exercise. These findings strengthen the hypothesis that these piRNAs may be functionally relevant in the context of physical activity.

The classification of piRNAs as germline-specific has been challenged by studies identifying piRNA-like small RNAs (piRNA-Ls) in somatic tissues. For example, Mei et al. (2015) [31] reported piRNAs in human lung tissue, showing interactions with p-ERM proteins, which regulate signal transduction and cytoskeleton dynamics. This discovery marked the first evidence of piRNA influencing cellular functions in mammalian somatic cells. Similarly, Rizzo et al. (2016) [33] identified piRNA-Ls in human liver cells, confirming PIWI-piRNA pathway activity and describing over 1600 piRNAs, including novel sequences.

While there is no confirmed PIWI interaction for piRNAs identified in our work yet, our findings collectively support the existence of somatic piRNAs and their potential functional roles. The identification of up to seven piRNAs previously described by Mei et al. (2015) [31] (hsa-piR-32835, hsa-piR-32874, hsa-piR-32956, hsa-piR-33036, hsa-piR-3304, hsa-piR-32941, hsa-piR-32886), underscores the pressing need for functional validation to confirm whether these sequences are true piRNAs or merely fragments of other non-coding RNAs (ncRNAs).

Thus, the evidence presented in this study and prior research underscores the potential of piRNAs as biomarkers in blood and somatic tissues. However, challenges such as multiple naming conventions and the lack of PIWI interaction data highlight the need for standardization and further research. Future studies should aim to confirm the role of piRNAs in training adaptation, validate their classification as genuine piRNAs, and explore their functional mechanisms of action in somatic cells.

One of the objectives of this study was to investigate whether baseline piRNA expression levels or changes in piRNA concentrations in response to exercise could serve as biomarkers for predicting individual training outcomes, such as improvements in V̇O2max (ΔV̇O2max). However, the weak and non-significant correlation between piRNA expression and ΔV̇O2max does not strongly support this hypothesis. It is important to note, however, that the small sample size and the limited number of piRNAs included in these analyses make it impossible to draw definitive conclusions.

In addition, despite these limitations, there were still a few piRNAs that demonstrated relatively strong correlations with ΔV̇O2max, suggesting a potential role in training adaptation: When assessing individual expression kinetics of the *n* = 13 selected piRNAs, most showed a gradual increase with exercise, of variable extent. Of those, piR-33074 appears particularly interesting, since its induction might also correlate with ΔV̇O2max. Furthermore, piR-12547 might be unique in a way, as it was immediately downregulated to almost zero in all participants in response to exercise, despite the fact that at rest, at least low and highly variable levels of this piRNA could be detected in all participants. Since baseline levels of this piRNA also showed good correlation with ΔV̇O2max and maximum expression levels were rather high, it might be a good candidate for a predictive biomarker of individual exercise adaptation. Finally, piR-11119, piR-33041, piR-33151, and piR-32886 displayed a more or less pronounced peak immediately after exercise. The latter two also showed correlation with ΔV̇O2max, both for baseline values and deltas, making them additional biomarker candidates for further testing. These findings highlight the need for further research to evaluate whether some specific piRNAs might be predictive of training adaptation, particularly in larger cohorts or using different selection criteria.

### Study Limitations

The primary limitation of this study is the small sample size, which restricted the analysis to simple statistical methods and prevented the drawing of definite, general conclusions. The study aimed to generate hypotheses regarding the potential roles of piRNAs as biomarkers for exercise and training regulation. The expression patterns identified now require validation through further investigations, involving larger cohorts and alternative quantification methods, such as qPCR.

In line with this, the number of subjects analyzed for each time point was different, further limiting the applicability of more refined statistical methods. Additionally, due to the pilot, hypothesis-generating character of our study, it was not possible to apply methods of correction for multiple testing/a false discovery rate adjustment, or a specific procedure for outlier exclusion. This will have to be performed in future, larger, confirmatory studies.

Furthermore, because of the small sample size, in the ΔV̇O2max correlation analysis, it was not possible to differentiate between subjects doing MICT or HIIT. Doing so would have resulted in groups of *n* = 3–4, which would not have been reasonable, especially when considering the interesting question of whether particular piRNA expression patterns not only correspond to training adaptation in general, but also to adaptation to a specific regimen. However, we will address this question in future studies, focusing on the set of potentially relevant piRNAs identified in the current study.

Additionally, our selection criteria for piRNAs were not entirely objective, as they were influenced by subjective assumptions and potential biases. Consequently, relevant piRNAs associated with training adaptations may have been overlooked during the selection process. Different selection criteria would likely have produced different results.

The study also did not compare baseline expression patterns with those measured during follow-up assessments, which could have provided valuable insights into long-term expression kinetics.

Furthermore, inconsistencies among existing piRNA databases posed a challenge. Some databases reference the same piRNA under different names, making it difficult to consolidate detailed information about the selected piRNAs. Thus, a comprehensive analysis of all available databases was not feasible, meaning that some relevant information on piRNAs and their nomenclature might not have been fully incorporated into this study.

Additionally, while differential piRNA expression patterns were observed, the exact mechanistic pathways underlying these, particularly the cell types responsible for their release, remain unclear. Thus, future work should explore the interplay between piRNA clusters and exercise-induced stimuli in somatic cells. In line with and complementary to this, analyzing piRNAs of extracellular vesicles in plasma might yield more specific results, eventually allowing mechanistic conclusions.

Finally, subsequent studies should focus on validating piRNA findings in larger, diverse cohorts to confirm reproducibility, elucidating the functional roles of specific piRNAs through in vitro and in vivo studies, exploring the temporal dynamics of piRNA expression across different exercise modalities, and integrating multi-omics approaches to connect piRNA changes with broader molecular and physiological adaptation markers.

## 4. Materials and Methods

### 4.1. Study Participants

Participants were part of the iReAct study, which investigated the biopsychosocial factors that influence individual adaptations to physical activity [1]. The study was registered in the German Clinical Trials Register on 12 June 2019 (DRKS00017446) and adhered to the ethical standards outlined by Harriss et al. (2019) [42]. Approval was obtained from the Ethics Committee of the Medical Faculty at the University of Tübingen on 22 January 2018 (reference number: 882/2017BO1), and all participants provided written informed consent prior to participation.

Individuals who reported engaging in less than 60 min of exercise per week during their leisure time—including activities such as team sports, aerobic exercise, or muscle strengthening—and had not followed a regular exercise routine in the past six months, were deemed eligible. Eligible candidates were invited to undergo a medical screening, which included a standard anamnesis and blood sample collection. To proceed with the diagnostic protocol, participants had to meet the following criteria: they were confirmed to be in good health based on the screening, had never recorded a body mass index (BMI) above 30 kg/m^2^ at any point in their life, and showed no signs of anemia or iron deficiency in their blood tests. The study recruited both male and female participants aged 20 to 40 years. Due to constraints imposed by the COVID-19 pandemic, the study cohort did not reach the initially planned *n* = 60, resulting in a final under-representation of males. Therefore, only data from female participants were included in the analysis described here, similar to previous studies on skeletal muscle miRNAs [14,43]. With regard to the subsequent training schedule on a bike ergometer, participants were randomly assigned to two groups—MICT-HIIT or HIIT-MICT—using a crossover design (Figure S1). The study was completed by 42 (M: *n* = 12, F: *n* = 30) participants, with 21 individuals included in each training sequence group [14]. For the piRNA analysis described here, owing to the screening character of this substudy, only plasma samples of *n* = 8 (‘at rest’) or 6 (all three time points; for details, see Section 4.4) female subjects were analyzed, *n* = 4/3 of which had been randomized to the MICT-HIIT and the other *n* = 4/3 to HIIT-MICT. The *n* = 6 subjects for which all three time points were evaluated were the same subgroup that had been analyzed in previous studies on skeletal muscle miRNAs [14,43]. For subjects’ characteristics, see Table 1.

### 4.2. Training Intervention Protocols

MICT involved 60 min of continuous cycling at the power output associated with 90% of the intensity corresponding to the first lactate threshold, while HIIT sessions consisted of a total of 43 min, including 10 min at 70% of the power output associated with the individual’s maximum heart rate (HRmax), followed by 4-min intervals of the power output associated with 90% HRmax, interspersed with 4-min recovery periods at 30 W, and concluding with a 5-min cool-down at 30 W. Lactate thresholds were determined using a segmented regression model with two breakpoints defined from the lactate-power output relationship, for details, see [13].

### 4.3. Diagnostics Blocks

Diagnostics were carried out before the training intervention (baseline) and after each training block (follow-ups FU1—after six weeks of training according to the MICT or HIIT protocol—and FU2—after switching to the respective other protocol and another six weeks of training, Figure S1), following the procedures described earlier [1]. In this manuscript, we focused solely on analyzing the effects of the initial training period, i.e., up to FU1. The assessments at each diagnostic time point included an extensive array of tests to capture a holistic biopsychosocial understanding of exercise. In particular, five different subprojects were carried out: (1) biographical mapping, (2) body image, (3) affective response, (4) physiological response, and (5) epigenetics, all aiming at a better understanding of individual responses to exercise. For details, see [1]. In this manuscript, we focused solely on piRNA patterns and their associations with exercise response and adaptation, i.e., defined aspects of subprojects (4) and (5) and their interplay.

### 4.4. Standardized Reference Training Protocol and Collection of Blood Samples

**Figure 5:**
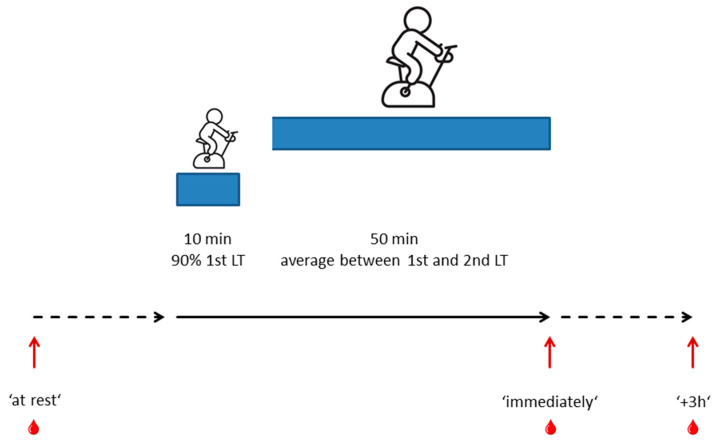
Standardized reference training. The reference training on a bicycle ergometer consisted of a 10-min warm-up at 90% of the intensity corresponding to the first lactate threshold, followed by 50 min cycling at the intensity halfway between the first and the second lactate thresholds. Blood samples were collected at three time points: 3 h before training (‘at rest’), directly after training (‘immediately’), and 3 h after training (‘+3 h’).

The standardized reference training was part of all three diagnostics blocks that were part of the study and was scheduled before the first and after the first and the second training blocks (baseline, FU1, and FU2, cf. Figure S1) [13]. It consisted of a 10-min warm-up period, consisting of cycling at an intensity corresponding to 90% of the first lactate threshold, followed by 50 min of cycling at a constant intensity corresponding to the midpoint between the first and second lactate thresholds ([13], Figure 5). Three blood samples were collected in the context of each reference training, one ‘at rest’, one post-exercise (‘immediately’), and one three hours post-exercise (‘+3 h’). In the context of this study, only baseline (i.e., pre-training) samples were analyzed.

### 4.5. Next-Generation Sequencing (NGS)

Twenty plasma samples were sent to GenXPro (Frankfurt, Germany) for NGS (‘at rest’ (*n* = 8), ‘immediately’ (*n* = 6), and ‘+3 h’ (*n* = 6)). There, after isolation of small RNAs using the Quick-cfRNA Serum & Plasma Kit (#R1059, Zymo Research, Irvine, CA, USA), the TrueQuant SmallRNA-Seq Kit for Ultra Low Input, GEL FREE, Single Tube Protocol Kit was employed to identify and quantify small RNAs. Normalization of individual piRNA concentrations was carried out according to Anders and Huber, 2010 [44].

### 4.6. Criteria Selection for Evaluation of Differentially Expressed piRNAs

A total of 17,304 different RNAs, such as piRNAs, miRNAs, lncRNAs, rRNAs, tRNAs, as well as protein-coding regions and others, were found. In the first step, the dataset was reduced to piRNA data and normalized piRNA data were regrouped for a better overview and for further calculations, grouping the samples by time taken and not by subject. This then left 411 piRNA species for further analysis. Next, the sum, mean, standard deviation, variance, fold change (average ‘immediately’/average ‘at rest’ and average ‘+3 h’/average ‘at rest’), and log2(fold change) (log2(fc)) were calculated. Subsequently, a decision on criteria to filter out the piRNA species that might be candidates for a more thorough analysis was made (Table 2).

Differentially expressed piRNA species were identified based on five criteria: (1) ‘fold change’ thresholds defined as a twofold or greater decrease (≤−2) or increase (≥2), between any of the three time points, (2) ‘log2(fc)’ thresholds defined as a twofold or greater decrease (≤−2) or increase (≥2), (3) ‘relative standard deviation’ in relation to the average, with values below 0.3 at at least one of the three time points analyzed, (4) selection of piRNAs with zero average normalized expression in either the first sample (‘at rest’), the second sample (‘immediately’), or the third sample (‘+3 h’) referred to as ‘zero expression’ and (5) the ‘increase/decrease’ requirement, which included piRNAs whose normalized average expression values consistently increased or decreased across all three samples (Table 2). For analysis of the first time point (‘at rest’), *n* = 8 samples were included, for the two later time points (‘immediately’ and ‘+3 h’), *n* = 6 samples were analyzed. The rationale for this approach was our hypothesis that for future applications as biomarkers, specifically baseline, ‘at rest’ markers might be useful. Thus, we decided to include two additional samples at this time point.

The lists of selected piRNAs found by the different criteria were then compared to see how the criteria overlap and which piRNAs met multiple selection criteria and which met only one (Table 2).

This evaluation represented the basis for deciding on criteria for candidate piRNA species for in-depth analysis (Table 2).

### 4.7. piRNA Selection

In a subsequent selection step, individual expression values of the pre-selected piRNA species for the eight participants were evaluated. To assess the consistency of expression patterns over time, line graphs were created to plot the normalized expression values of each piRNA at the three time points: ‘at rest’, ‘immediately’, and ‘+3 h’ for each participant. Additionally, bar graphs depicting the average normalized expression levels along with standard deviations were generated.

The graphs were analyzed to identify comparable results across all or most participants, such as a general increase or decrease in expression. A particular piRNA was considered suitable for further analysis if its expression pattern was consistent, meaning no more than one participant displayed a markedly different trend (e.g., a sharp decrease while others showed an increase). Such an expression pattern was defined as ‘homogeneous’ or ‘uniform’.

If no consistent pattern was evident, the respective piRNA species were excluded from further analysis. Similarly, piRNAs were removed if two or more participants exhibited no detectable expression, especially when the remaining data lacked a clear trend. Finally, piRNAs were excluded if they showed predominantly zero values (i.e., three or more participants had zero expression across all time points) or if overall expression values were very low (below a threshold of 1).

### 4.8. piRNA Characterization

The PIWI-interacting RNA (piRNA) Database (piRNAdb) [30] was consulted to gather additional information about the selected piRNA species. In particular, sequences were examined for typical piRNA features, such as a length of 24–35 nucleotides [24], a 5′ uridine bias, or an adenine bias at the tenth nucleotide position [17].

### 4.9. Correlation Analysis

Statistical analyses included assessing the correlation between piRNA expression and changes in V̇O2max (delta of maximal oxygen uptake; ΔV̇O2max). ΔV̇O2max was calculated as the difference between baseline V̇O2max and FU1 V̇O2max. Spearman’s rank correlation was applied for the analysis. The ranking required for the correlation analysis was performed using the Excel function RANK.AVG, and correlations were calculated using the CORREL function.

### 4.10. Data Visualization

Python version 3.12.0, and Jupyter Notebook 7.3 were used to perform data visualization.

## 5. Conclusions

While the precise role of piRNAs in human somatic cells remains incompletely understood, our findings provide preliminary evidence supporting their potential as biomarkers in the context of exercise. Our data suggest that they can be assessed from the circulation of healthy subjects and display characteristic changes in expression when challenged by exercise. Furthermore, our results indicate that baseline, as well as differential piRNA expression in response to exercise, might correlate with exercise adaptation, suggesting that piRNA patterns might be candidates for potential biomarkers in training optimization and monitoring. Validation in larger cohorts, using complementary techniques such as qPCR, however, is essential to confirm these expression patterns. Furthermore, additional studies are needed to elucidate the mechanistic roles of piRNAs in exercise physiology and to identify other potentially relevant piRNAs that may have been missed. Thus, this work lays the groundwork for further exploration of the biological and diagnostic significance of piRNAs in training and exercise adaptation.

## Figures and Tables

**Table 1:** Participant demographics and performance metrics. The table displays individual identifiers (ID), experimental group assignment (MICT-HIIT or HIIT-MICT), gender, age, baseline weight, height, body mass index (BMI), and relative V̇O2max values at baseline and first follow-up (FU1). For two participants, IR0015 and IR0046, labelled in gray, piRNA patterns were only analyzed in ‘at rest’ blood samples.

ID	Group	Gender	Age	Baseline Weight [kg]	Baseline Height [cm]	Baseline BMI [kg/m^2^]	Baseline Absolute V̇O_2max_ [L/min]	Baseline Relative V̇O_2max_ [L/kgxmin]	FU1 Absolute V̇O_2max_ [L/min]	FU1 Relative V̇O_2max_ [L/kgxmin]
IR0005	MICT-HIIT	Female	22	61.2	167.5	21.80	2.03	33.2	2.39	38.9
IR0010	MICT-HIIT	Female	21	66.5	163	25.03	2.04	30.65	2.23	34.02
IR0030	MICT-HIIT	Female	29	60.9	159	24.09	2.04	33.55	2.19	36.24
IR0046	MICT-HIIT	Female	26	66.2	174	21.8	2.03	30.71	2.28	34.13
IR0008	HIIT-MICT	Female	20	59.3	164.5	21.9	1.95	32.82	2.28	37.62
IR0012	HIIT-MICT	Female	21	68.9	173.5	22.8	2.01	29.15	2.25	33.16
IR0015	HIIT-MICT	Female	28	61.7	174.2	20.3	1.97	31.93	2.30	36.62
IR0042	HIIT-MICT	Female	27	57.6	160.4	22.3	1.77	30.8	1.94	34.97

**Table 2:** Overview of piRNA selection and filtering process via small RNA NGS sequencing. Initial sequencing identified 17,304 RNAs, including piRNAs, miRNAs, lncRNAs, rRNA, tRNA, and protein-coding regions. The dataset was reduced to 411 piRNAs after normalization and regrouping by sampling time. For further analysis, several statistical metrics were calculated, including sum, mean, standard deviation, variance, fold change, and log2(fold change). A multi-step filtering process was applied, involving criteria such as log2(fold change) (‘log2(fc)’), fold change, relative standard deviation, zero expression, and expression pattern (increase or decrease across samples; ‘increase/decrease’). The selected piRNAs were compared across these criteria to identify candidates for deeper analysis.

**1st**
**Reduce data set to piRNA data**
**2nd**
**Calculate descriptive statistics**
sum
mean
standard deviation
variance
fold change
log2(fc)
**3rd**
**Testing several selection criteria**
relative standard deviation
log_2_(fc)
zero expression
increase/decrease
fold change
**4th**
**Comparing piRNA lists of different criteria**
**5th**
**Deciding on criteria**

## Data Availability

All data are available from the authors upon reasonable request.

## Supplementary Materials

The following supporting information can be downloaded at https://www.mdpi.com/article/10.3390/ncrna11030046/s1, Table S1. Compilation of piRNA count distributions across various selection criteria. The table displays the total number of piRNAs for each of the following parameters: ‘log2(fc)’, ‘fold change’, ‘relative standard deviation’, ‘zero expression’, and ‘increase/decrease’. Table S2. Analysis of piRNA counts across multiple selection criteria. This table presents the number of piRNAs shared among various combinations of the following parameters: ‘log2(fc)’, ‘fold change’, ‘relative standard deviation’, ‘zero expression’, and ‘increase/decrease’. Both single-criterion and multi-criteria datasets are highlighted, accompanied by the corresponding piRNA identification labels (e.g., ‘hs-piR-xxx’). Table S3. Overview of selected piRNAs referenced in the piRNAdb database. Three key studies (Girard et al., 2006 [32]; Mei et al., 2015 [31]; Rizzo et al., 2016 [33]) identified specific piRNAs through immunoprecipitation (testis tissue) and small RNA sequencing (lung and liver tissue). The selected piRNAs exhibited characteristic length and nucleotide features, including 5′ uridine for antisense piRNAs and adenine at or near the 10th nucleotide for sense piRNAs. Most piRNAs showed increased expression during exercise, with hsa-piR-33036 and hsa-piR-25111 displaying the most pronounced and consistent upregulation. Conversely, hsa-piR-33151 and hsa-piR-12547 showed an overall decrease in expression. Table S4. Correlation analysis between piRNA expression and V̇O2max. To investigate potential correlations with exercise-induced changes, average piRNA expression values were correlated with ΔV̇O2max values of the participants. Additionally, the differences in piRNA expression between the ‘+3 h’ and ‘at rest’ time points (Δ expression values) were calculated and correlated with ΔV̇O2max. ΔV̇O2max was determined by subtracting baseline V̇O2max from follow-up V̇O2max (FU1). Figure S1. iReact Study design and timeline. Adapted from Thiel et al., 2020 [1]. The study consisted of three main phases: baseline (week 1), training (weeks 2–7 and weeks 9–14), and follow-up (weeks 8 and 15). During the baseline phase, activity monitoring and biophysical characteristics were assessed, followed by blood sampling. The training phase included two exercise protocols: moderate-intensity continuous training (MICT) and high-intensity interval training (HIIT), with participants undergoing 60-min sessions at 90% of the first lactate threshold (LT) for MICT or 4 × 4-min intervals at 90% of maximum heart rate (HRmax) for HIIT. Follow-up assessments were conducted post-training at weeks 8 and 15, including repeated activity monitoring, biophysical assessments, and blood sampling. Muscle biopsies and in situ exercise experiences were performed at designated time points, as indicated by the legend.

## Abbreviations

PIWI	A family of proteins interacting with piRNAs
piRNA	PIWI-interacting RNA
piRNAdb	PIWI-interacting RNA Database
piRBase	A piRNA database with different naming conventions
HIIT	High-intensity interval training
MICT	Moderate-intensity continuous training
rRNA	Ribosomal RNA
sncRNA	Small non-coding RNA
miRNA	MicroRNA
lncRNA	Long non-coding RNA
tRNA	Transfer RNA
p-ERM	Protein-ERM (Ezrin, Radixin, Moesin) complex, related to cytoskeleton regulation
piRNA-Ls	piRNA-like small RNAs
hsa-piR-xxx	Human piRNA identification (e.g., hsa-piR-1677, hsa-piR-11119)
RER	Respiratory exchange ratio
NGS	Next-generation sequencing
HRmax	Maximum heart rate
V̇O2max	Maximal oxygen uptake
log2(fc)	Logarithmic base 2 fold change
qPCR	Quantitative polymerase chain reaction
NCBI	National Center for Biotechnology Information
