# Supplementary material for: Patterns of Circulating piRNAs in the Context of a Single Bout of Exercise: Potential Biomarkers of Exercise-Induced Adaptation?
Source: Noncoding RNA. 2025 Jun 16;11(3):46. doi: 10.3390/ncrna11030046 (PMC12195705; doi:10.3390/ncrna11030046)
Supplement: Supplementary file 1 [file ncrna-11-00046-s001.zip › Table S3 revised.pptx]

## Slide 1
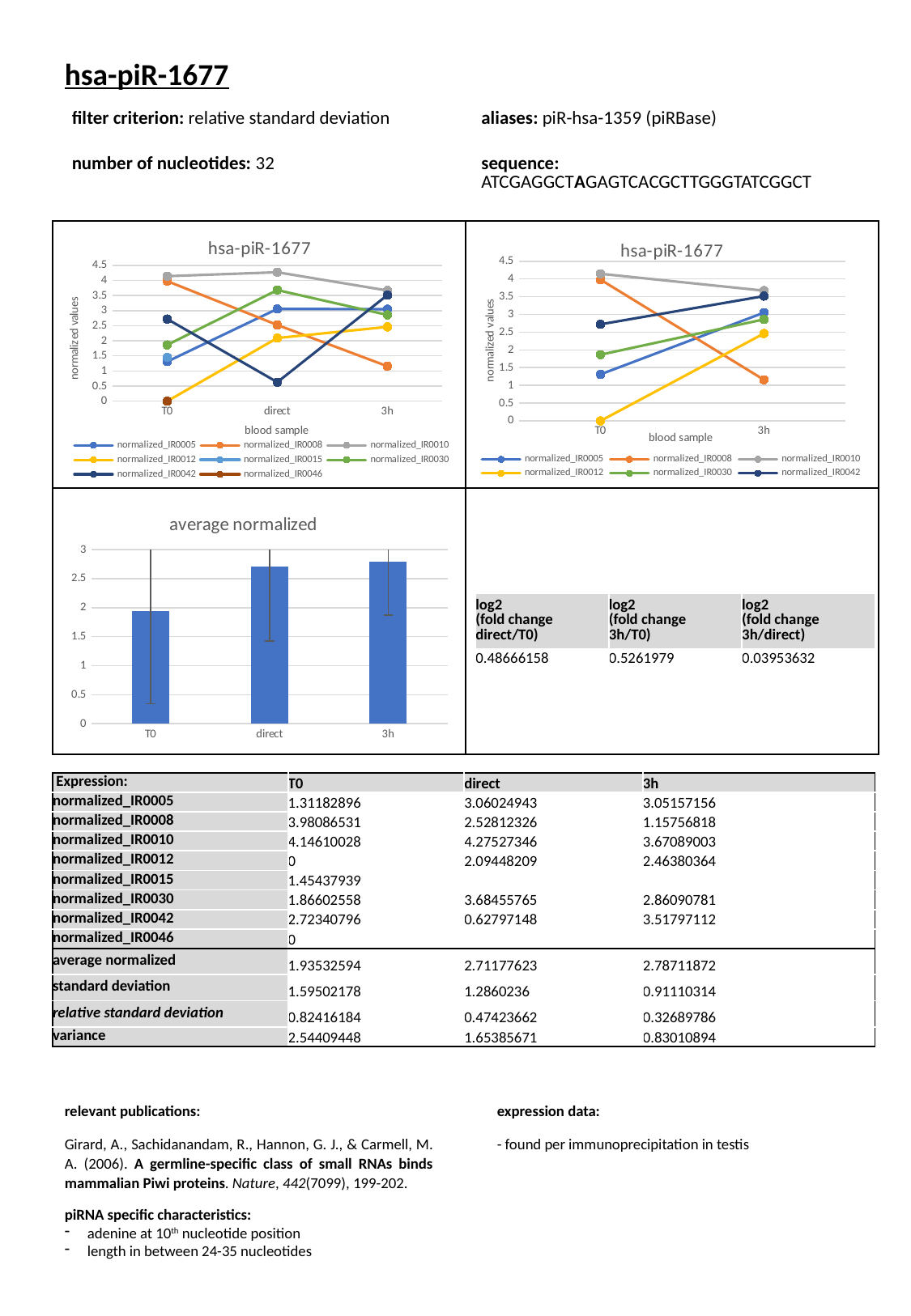

hsa-piR-1677
| filter criterion: relative standard deviation | aliases: piR-hsa-1359 (piRBase) |
| --- | --- |
| number of nucleotides: 32 | sequence: ATCGAGGCTAGAGTCACGCTTGGGTATCGGCT |
[unsupported chart]
### Chart:
| Category | normalized_IR0005 | normalized_IR0008 | normalized_IR0010 | normalized_IR0012 | normalized_IR0030 | normalized_IR0042 |
|---|---|---|---|---|---|---|
| T0 | 1.311828958493076 | 3.980865307422324 | 4.146100281727515 | 0.0 | 1.866025583210745 | 2.723407963789568 |
| 3h | 3.051571559353067 | 1.157568180765847 | 3.670890025641168 | 2.463803644704732 | 2.860907811823217 | 3.517971115698155 |
### Chart: average normalized
| Category | Durchschnitt_normalized |
|---|---|
| T0 | 1.9353259356881884 |
| direct | 2.711776227881487 |
| 3h | 2.787118722997698 || log2 (fold change direct/T0) | log2 (fold change 3h/T0) | log2 (fold change 3h/direct) |
| --- | --- | --- |
| 0.48666158 | 0.5261979 | 0.03953632 |
| Expression: | T0 | direct | 3h |
| --- | --- | --- | --- |
| normalized\_IR0005 | 1.31182896 | 3.06024943 | 3.05157156 |
| normalized\_IR0008 | 3.98086531 | 2.52812326 | 1.15756818 |
| normalized\_IR0010 | 4.14610028 | 4.27527346 | 3.67089003 |
| normalized\_IR0012 | 0 | 2.09448209 | 2.46380364 |
| normalized\_IR0015 | 1.45437939 | | |
| normalized\_IR0030 | 1.86602558 | 3.68455765 | 2.86090781 |
| normalized\_IR0042 | 2.72340796 | 0.62797148 | 3.51797112 |
| normalized\_IR0046 | 0 | | |
| average normalized | 1.93532594 | 2.71177623 | 2.78711872 |
| standard deviation | 1.59502178 | 1.2860236 | 0.91110314 |
| relative standard deviation | 0.82416184 | 0.47423662 | 0.32689786 |
| variance | 2.54409448 | 1.65385671 | 0.83010894 |
relevant publications:
Girard, A., Sachidanandam, R., Hannon, G. J., & Carmell, M. A. (2006). A germline-specific class of small RNAs binds mammalian Piwi proteins. Nature, 442(7099), 199-202.
piRNA specific characteristics:
adenine at 10th nucleotide position
length in between 24-35 nucleotides
expression data:
- found per immunoprecipitation in testis

## Slide 2
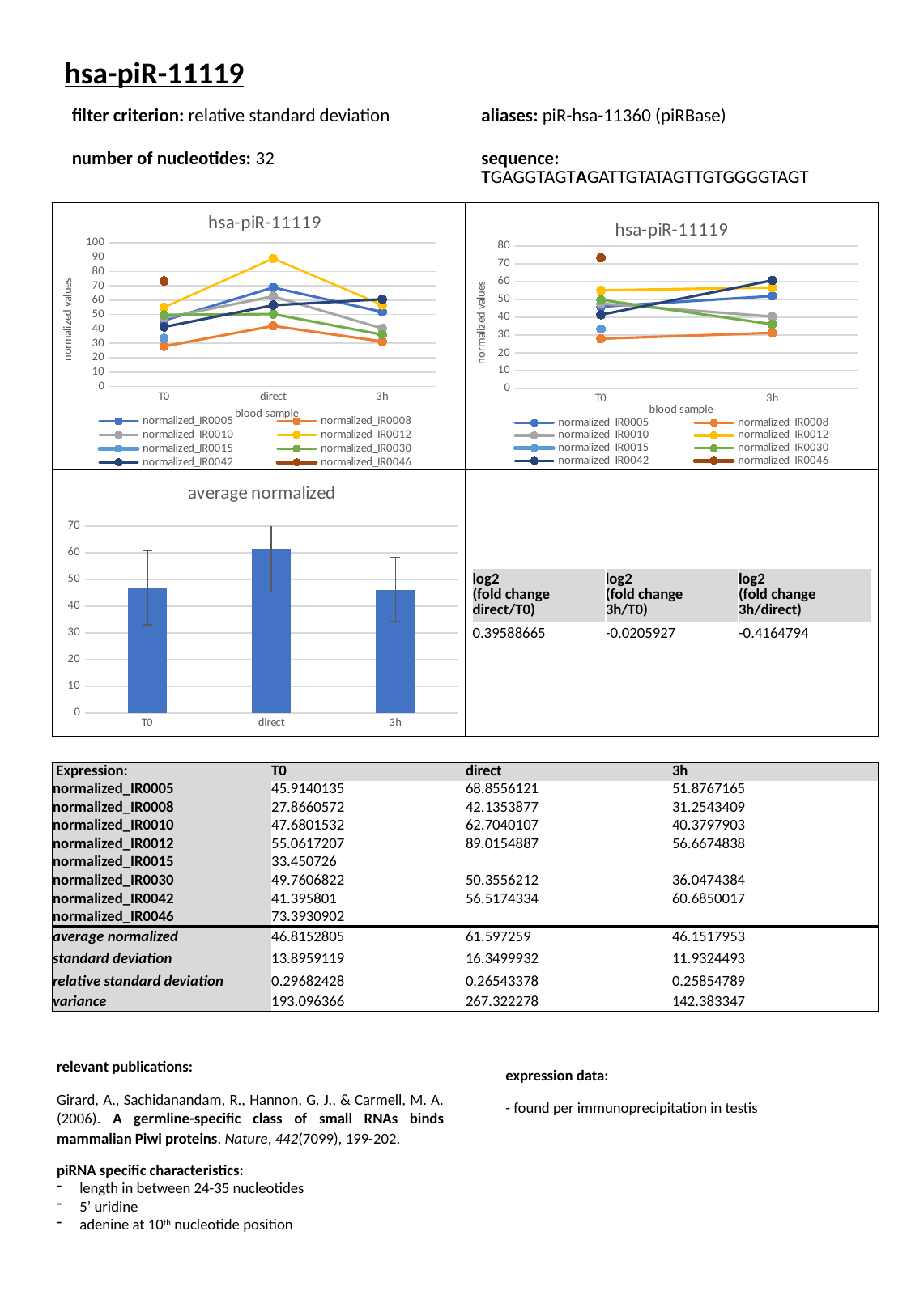

hsa-piR-11119
| filter criterion: relative standard deviation | aliases: piR-hsa-11360 (piRBase) |
| --- | --- |
| number of nucleotides: 32 | sequence: TGAGGTAGTAGATTGTATAGTTGTGGGGTAGT |
[unsupported chart]
[unsupported chart]
### Chart: average normalized
| Category | Durchschnitt_normalized |
|---|---|
| T0 | 46.81528051814696 |
| direct | 61.597258978722074 |
| 3h | 46.15179527911789 || log2 (fold change direct/T0) | log2 (fold change 3h/T0) | log2 (fold change 3h/direct) |
| --- | --- | --- |
| 0.39588665 | -0.0205927 | -0.4164794 |
| Expression: | T0 | direct | 3h |
| --- | --- | --- | --- |
| normalized\_IR0005 | 45.9140135 | 68.8556121 | 51.8767165 |
| normalized\_IR0008 | 27.8660572 | 42.1353877 | 31.2543409 |
| normalized\_IR0010 | 47.6801532 | 62.7040107 | 40.3797903 |
| normalized\_IR0012 | 55.0617207 | 89.0154887 | 56.6674838 |
| normalized\_IR0015 | 33.450726 | | |
| normalized\_IR0030 | 49.7606822 | 50.3556212 | 36.0474384 |
| normalized\_IR0042 | 41.395801 | 56.5174334 | 60.6850017 |
| normalized\_IR0046 | 73.3930902 | | |
| average normalized | 46.8152805 | 61.597259 | 46.1517953 |
| standard deviation | 13.8959119 | 16.3499932 | 11.9324493 |
| relative standard deviation | 0.29682428 | 0.26543378 | 0.25854789 |
| variance | 193.096366 | 267.322278 | 142.383347 |
relevant publications:
Girard, A., Sachidanandam, R., Hannon, G. J., & Carmell, M. A. (2006). A germline-specific class of small RNAs binds mammalian Piwi proteins. Nature, 442(7099), 199-202.
piRNA specific characteristics:
length in between 24-35 nucleotides
5’ uridine
adenine at 10th nucleotide position
expression data:
- found per immunoprecipitation in testis

## Slide 3
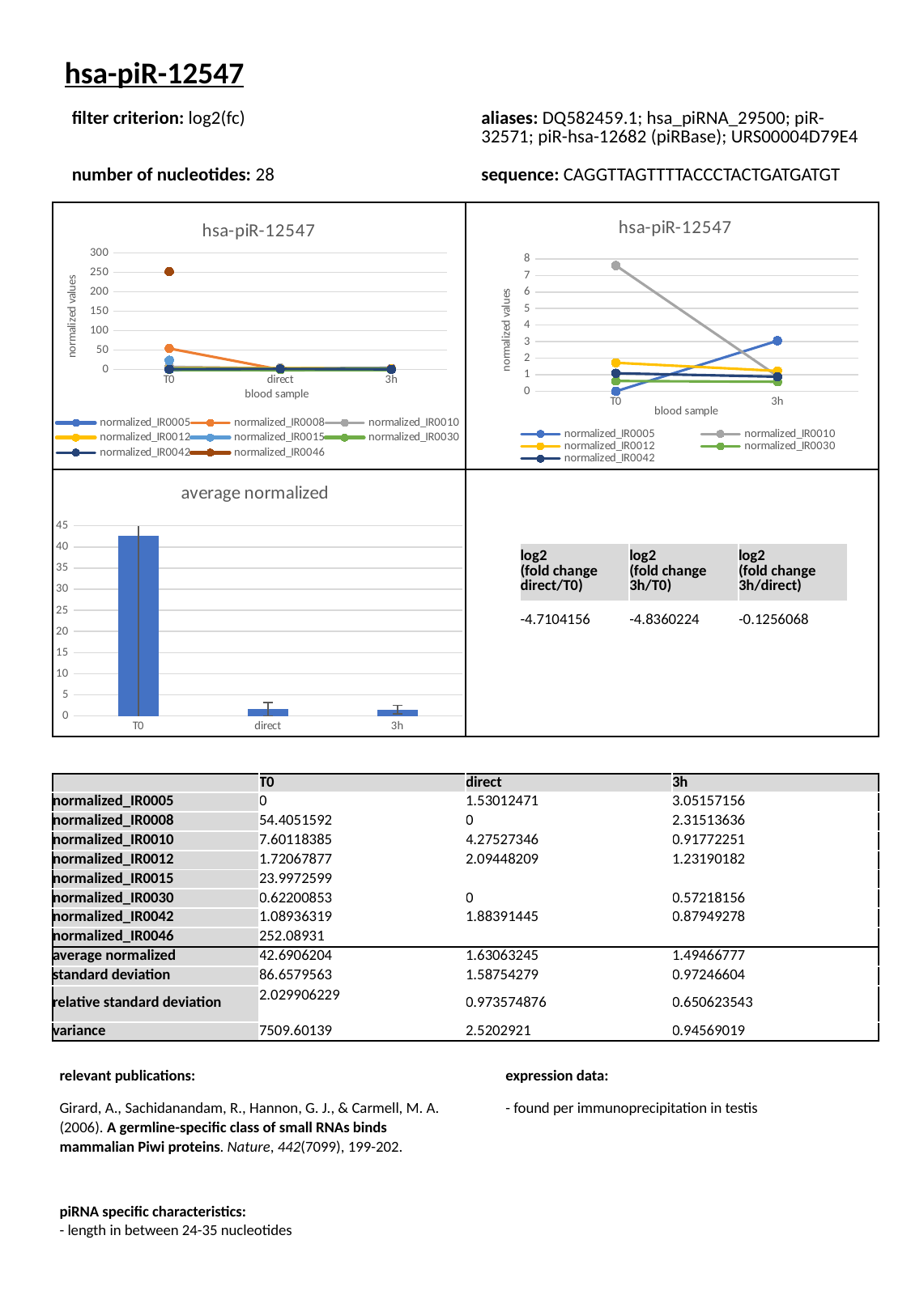

hsa-piR-12547
| filter criterion: log2(fc) | aliases: DQ582459.1; hsa\_piRNA\_29500; piR-32571; piR-hsa-12682 (piRBase); URS00004D79E4 |
| --- | --- |
| number of nucleotides: 28 | sequence: CAGGTTAGTTTTACCCTACTGATGATGT |
### Chart:
| Category | normalized_IR0005 | normalized_IR0010 | normalized_IR0012 | normalized_IR0030 | normalized_IR0042 |
|---|---|---|---|---|---|
| T0 | 0.0 | 7.601183849833776 | 1.720678773362516 | 0.6220085277369152 | 1.089363185515827 |
| 3h | 3.051571559353067 | 0.9177225064102916 | 1.231901822352366 | 0.5721815623646432 | 0.8794927789245387 |
[unsupported chart]
### Chart: average normalized
| Category | Durchschnitt_normalized |
|---|---|
| T0 | 42.690620413109606 |
| direct | 1.6306324507687127 |
| 3h | 1.4946677651561 || log2 (fold change direct/T0) | log2 (fold change 3h/T0) | log2 (fold change 3h/direct) |
| --- | --- | --- |
| -4.7104156 | -4.8360224 | -0.1256068 |
| | T0 | direct | 3h |
| --- | --- | --- | --- |
| normalized\_IR0005 | 0 | 1.53012471 | 3.05157156 |
| normalized\_IR0008 | 54.4051592 | 0 | 2.31513636 |
| normalized\_IR0010 | 7.60118385 | 4.27527346 | 0.91772251 |
| normalized\_IR0012 | 1.72067877 | 2.09448209 | 1.23190182 |
| normalized\_IR0015 | 23.9972599 | | |
| normalized\_IR0030 | 0.62200853 | 0 | 0.57218156 |
| normalized\_IR0042 | 1.08936319 | 1.88391445 | 0.87949278 |
| normalized\_IR0046 | 252.08931 | | |
| average normalized | 42.6906204 | 1.63063245 | 1.49466777 |
| standard deviation | 86.6579563 | 1.58754279 | 0.97246604 |
| relative standard deviation | 2.029906229 | 0.973574876 | 0.650623543 |
| variance | 7509.60139 | 2.5202921 | 0.94569019 |
relevant publications:
Girard, A., Sachidanandam, R., Hannon, G. J., & Carmell, M. A. (2006). A germline-specific class of small RNAs binds mammalian Piwi proteins. Nature, 442(7099), 199-202.
piRNA specific characteristics:
- length in between 24-35 nucleotides
expression data:
- found per immunoprecipitation in testis

## Slide 4
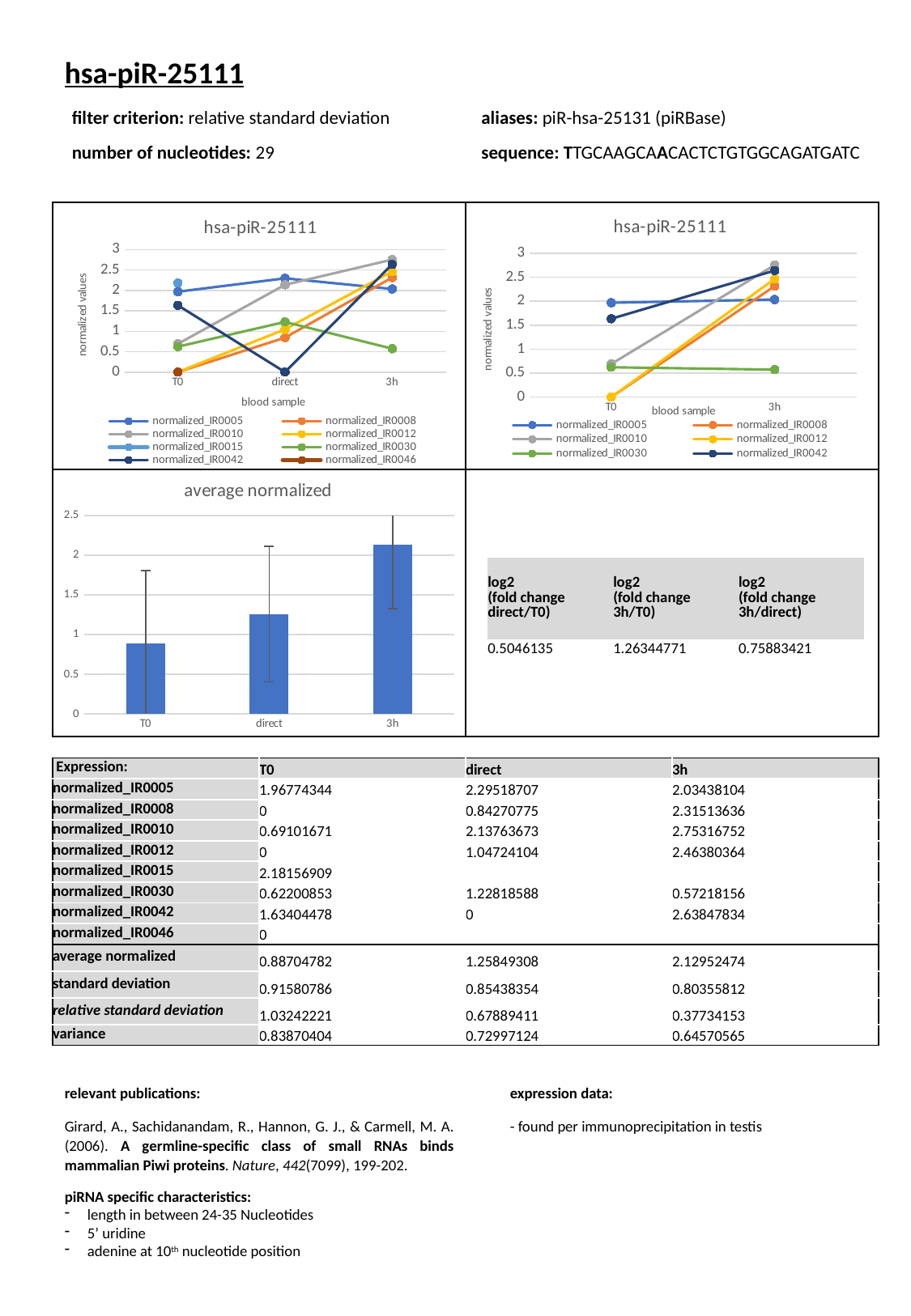

hsa-piR-25111
| filter criterion: relative standard deviation | aliases: piR-hsa-25131 (piRBase) |
| --- | --- |
| number of nucleotides: 29 | sequence: TTGCAAGCAACACTCTGTGGCAGATGATC |
### Chart:
| Category | normalized_IR0005 | normalized_IR0008 | normalized_IR0010 | normalized_IR0012 | normalized_IR0030 | normalized_IR0042 |
|---|---|---|---|---|---|---|
| T0 | 1.967743437739614 | 0.0 | 0.6910167136212524 | 0.0 | 0.6220085277369152 | 1.634044778273741 |
| 3h | 2.034381039568711 | 2.315136361531694 | 2.753167519230875 | 2.463803644704732 | 0.5721815623646432 | 2.638478336773616 |
[unsupported chart]
### Chart: average normalized
| Category | Durchschnitt_normalized |
|---|---|
| T0 | 0.8870478179581177 |
| direct | 1.2584930799348282 |
| 3h | 2.129524744029045 || log2 (fold change direct/T0) | log2 (fold change 3h/T0) | log2 (fold change 3h/direct) |
| --- | --- | --- |
| 0.5046135 | 1.26344771 | 0.75883421 |
| Expression: | T0 | direct | 3h |
| --- | --- | --- | --- |
| normalized\_IR0005 | 1.96774344 | 2.29518707 | 2.03438104 |
| normalized\_IR0008 | 0 | 0.84270775 | 2.31513636 |
| normalized\_IR0010 | 0.69101671 | 2.13763673 | 2.75316752 |
| normalized\_IR0012 | 0 | 1.04724104 | 2.46380364 |
| normalized\_IR0015 | 2.18156909 | | |
| normalized\_IR0030 | 0.62200853 | 1.22818588 | 0.57218156 |
| normalized\_IR0042 | 1.63404478 | 0 | 2.63847834 |
| normalized\_IR0046 | 0 | | |
| average normalized | 0.88704782 | 1.25849308 | 2.12952474 |
| standard deviation | 0.91580786 | 0.85438354 | 0.80355812 |
| relative standard deviation | 1.03242221 | 0.67889411 | 0.37734153 |
| variance | 0.83870404 | 0.72997124 | 0.64570565 |
relevant publications:
Girard, A., Sachidanandam, R., Hannon, G. J., & Carmell, M. A. (2006). A germline-specific class of small RNAs binds mammalian Piwi proteins. Nature, 442(7099), 199-202.
piRNA specific characteristics:
length in between 24-35 Nucleotides
5’ uridine
adenine at 10th nucleotide position
expression data:
- found per immunoprecipitation in testis

## Slide 5
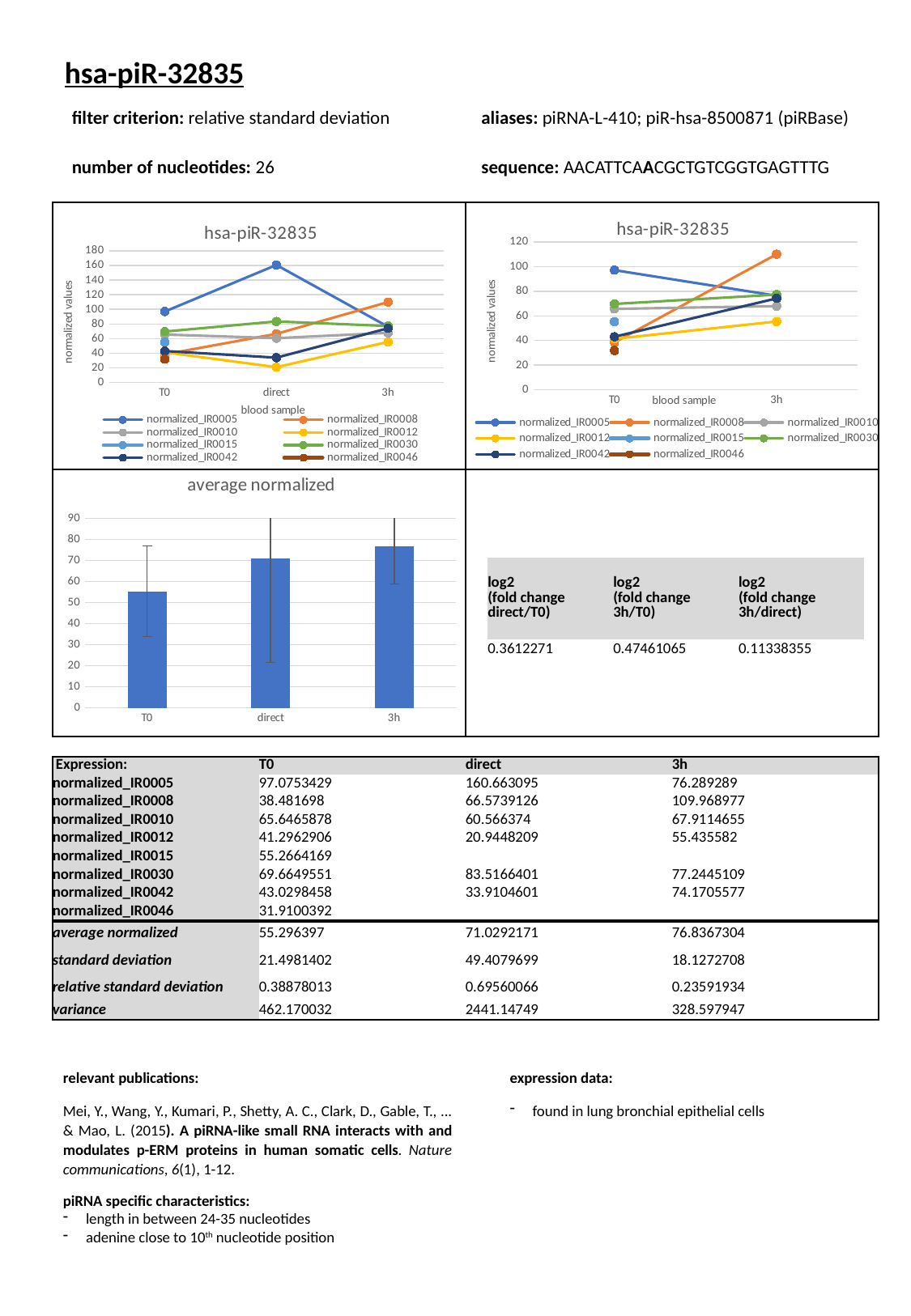

hsa-piR-32835
| filter criterion: relative standard deviation | aliases: piRNA-L-410; piR-hsa-8500871 (piRBase) |
| --- | --- |
| number of nucleotides: 26 | sequence: AACATTCAACGCTGTCGGTGAGTTTG |
[unsupported chart]
[unsupported chart]
### Chart: average normalized
| Category | Durchschnitt_normalized |
|---|---|
| T0 | 55.296397032446336 |
| direct | 71.02921707656779 |
| 3h | 76.83673037422164 || log2 (fold change direct/T0) | log2 (fold change 3h/T0) | log2 (fold change 3h/direct) |
| --- | --- | --- |
| 0.3612271 | 0.47461065 | 0.11338355 |
| Expression: | T0 | direct | 3h |
| --- | --- | --- | --- |
| normalized\_IR0005 | 97.0753429 | 160.663095 | 76.289289 |
| normalized\_IR0008 | 38.481698 | 66.5739126 | 109.968977 |
| normalized\_IR0010 | 65.6465878 | 60.566374 | 67.9114655 |
| normalized\_IR0012 | 41.2962906 | 20.9448209 | 55.435582 |
| normalized\_IR0015 | 55.2664169 | | |
| normalized\_IR0030 | 69.6649551 | 83.5166401 | 77.2445109 |
| normalized\_IR0042 | 43.0298458 | 33.9104601 | 74.1705577 |
| normalized\_IR0046 | 31.9100392 | | |
| average normalized | 55.296397 | 71.0292171 | 76.8367304 |
| standard deviation | 21.4981402 | 49.4079699 | 18.1272708 |
| relative standard deviation | 0.38878013 | 0.69560066 | 0.23591934 |
| variance | 462.170032 | 2441.14749 | 328.597947 |
relevant publications:
Mei, Y., Wang, Y., Kumari, P., Shetty, A. C., Clark, D., Gable, T., ... & Mao, L. (2015). A piRNA-like small RNA interacts with and modulates p-ERM proteins in human somatic cells. Nature communications, 6(1), 1-12.
piRNA specific characteristics:
length in between 24-35 nucleotides
adenine close to 10th nucleotide position
expression data:
found in lung bronchial epithelial cells

## Slide 6
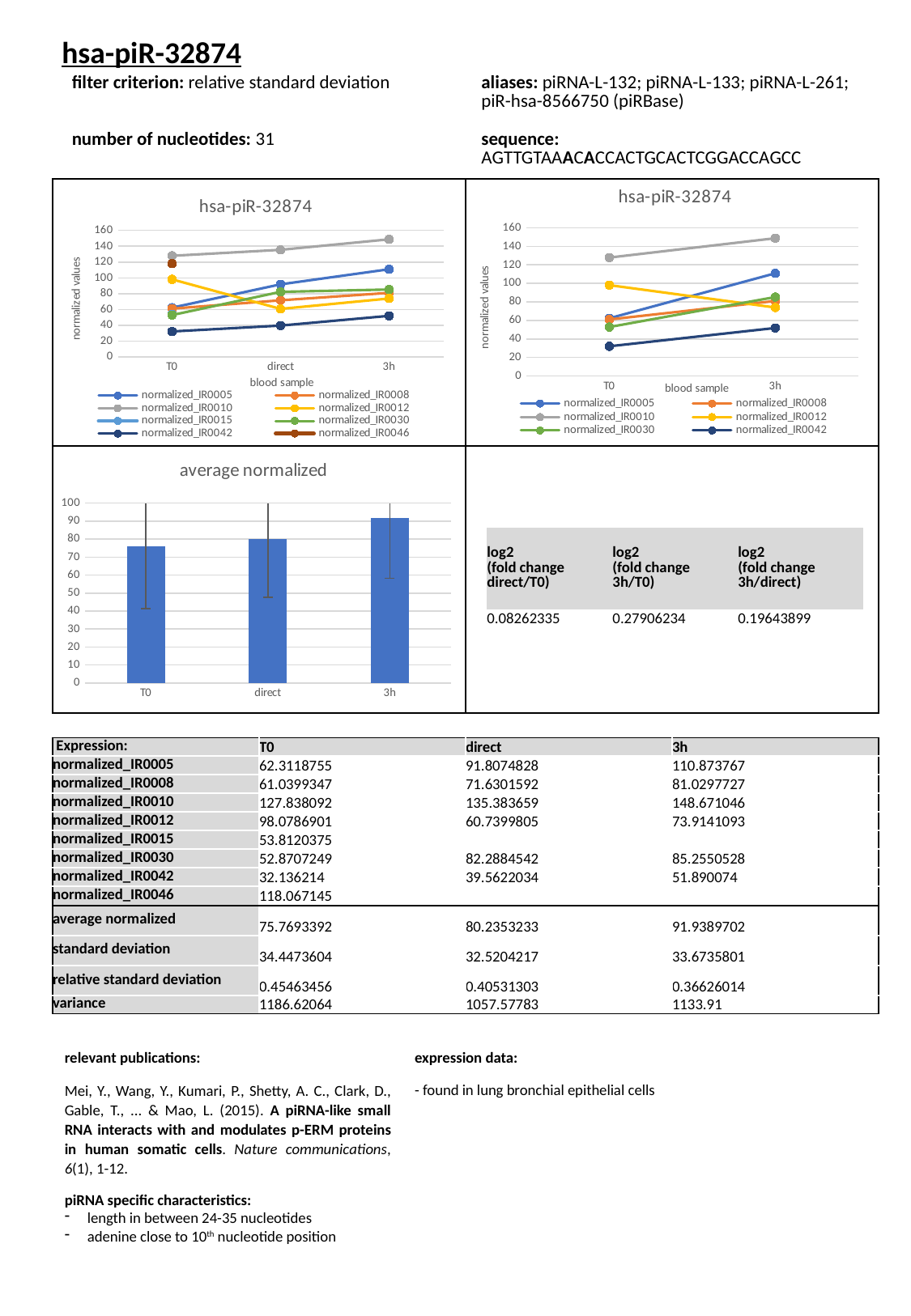

hsa-piR-32874
| filter criterion: relative standard deviation | aliases: piRNA-L-132; piRNA-L-133; piRNA-L-261; piR-hsa-8566750 (piRBase) |
| --- | --- |
| number of nucleotides: 31 | sequence: AGTTGTAAACACCACTGCACTCGGACCAGCC |
### Chart:
| Category | normalized_IR0005 | normalized_IR0008 | normalized_IR0010 | normalized_IR0012 | normalized_IR0030 | normalized_IR0042 |
|---|---|---|---|---|---|---|
| T0 | 62.31187552842111 | 61.03993471380896 | 127.8380920199317 | 98.0786900816634 | 52.8707248576378 | 32.1362139727169 |
| 3h | 110.8737666564948 | 81.02977265360929 | 148.6710460384673 | 73.91410934114195 | 85.25505279233185 | 51.89007395654778 |
[unsupported chart]
### Chart: average normalized
| Category | Durchschnitt_normalized |
|---|---|
| T0 | 75.76933921757569 |
| direct | 80.23532325398617 |
| 3h | 91.93897023976551 || log2 (fold change direct/T0) | log2 (fold change 3h/T0) | log2 (fold change 3h/direct) |
| --- | --- | --- |
| 0.08262335 | 0.27906234 | 0.19643899 |
| Expression: | T0 | direct | 3h |
| --- | --- | --- | --- |
| normalized\_IR0005 | 62.3118755 | 91.8074828 | 110.873767 |
| normalized\_IR0008 | 61.0399347 | 71.6301592 | 81.0297727 |
| normalized\_IR0010 | 127.838092 | 135.383659 | 148.671046 |
| normalized\_IR0012 | 98.0786901 | 60.7399805 | 73.9141093 |
| normalized\_IR0015 | 53.8120375 | | |
| normalized\_IR0030 | 52.8707249 | 82.2884542 | 85.2550528 |
| normalized\_IR0042 | 32.136214 | 39.5622034 | 51.890074 |
| normalized\_IR0046 | 118.067145 | | |
| average normalized | 75.7693392 | 80.2353233 | 91.9389702 |
| standard deviation | 34.4473604 | 32.5204217 | 33.6735801 |
| relative standard deviation | 0.45463456 | 0.40531303 | 0.36626014 |
| variance | 1186.62064 | 1057.57783 | 1133.91 |
relevant publications:
Mei, Y., Wang, Y., Kumari, P., Shetty, A. C., Clark, D., Gable, T., ... & Mao, L. (2015). A piRNA-like small RNA interacts with and modulates p-ERM proteins in human somatic cells. Nature communications, 6(1), 1-12.
piRNA specific characteristics:
length in between 24-35 nucleotides
adenine close to 10th nucleotide position
expression data:
- found in lung bronchial epithelial cells

## Slide 7
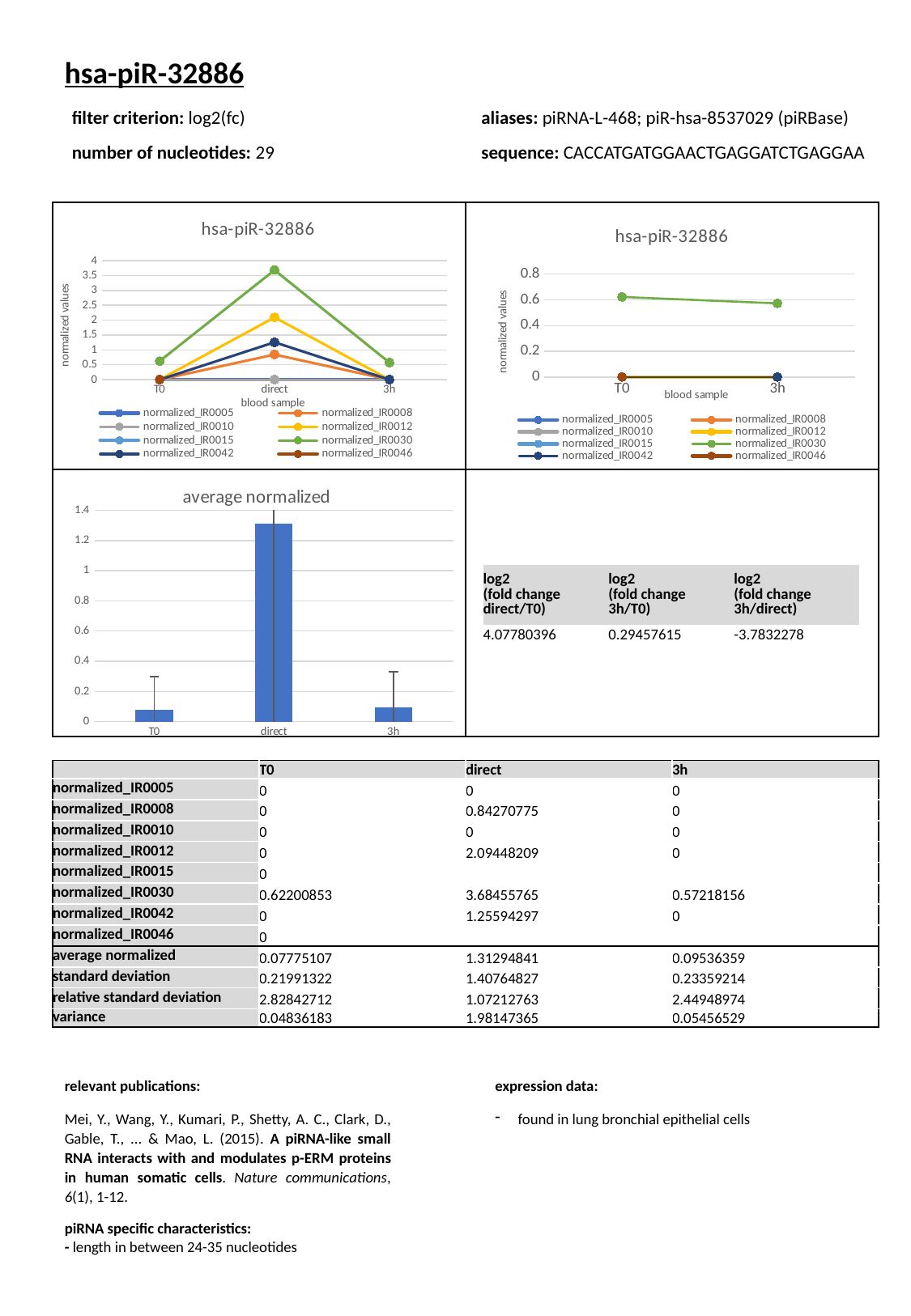

hsa-piR-32886
| filter criterion: log2(fc) | aliases: piRNA-L-468; piR-hsa-8537029 (piRBase) |
| --- | --- |
| number of nucleotides: 29 | sequence: CACCATGATGGAACTGAGGATCTGAGGAA |
[unsupported chart]
[unsupported chart]
### Chart: average normalized
| Category | Durchschnitt_normalized |
|---|---|
| T0 | 0.0777510659671144 |
| direct | 1.3129484095568118 |
| 3h | 0.09536359372744053 || log2 (fold change direct/T0) | log2 (fold change 3h/T0) | log2 (fold change 3h/direct) |
| --- | --- | --- |
| 4.07780396 | 0.29457615 | -3.7832278 |
| | T0 | direct | 3h |
| --- | --- | --- | --- |
| normalized\_IR0005 | 0 | 0 | 0 |
| normalized\_IR0008 | 0 | 0.84270775 | 0 |
| normalized\_IR0010 | 0 | 0 | 0 |
| normalized\_IR0012 | 0 | 2.09448209 | 0 |
| normalized\_IR0015 | 0 | | |
| normalized\_IR0030 | 0.62200853 | 3.68455765 | 0.57218156 |
| normalized\_IR0042 | 0 | 1.25594297 | 0 |
| normalized\_IR0046 | 0 | | |
| average normalized | 0.07775107 | 1.31294841 | 0.09536359 |
| standard deviation | 0.21991322 | 1.40764827 | 0.23359214 |
| relative standard deviation | 2.82842712 | 1.07212763 | 2.44948974 |
| variance | 0.04836183 | 1.98147365 | 0.05456529 |
relevant publications:
Mei, Y., Wang, Y., Kumari, P., Shetty, A. C., Clark, D., Gable, T., ... & Mao, L. (2015). A piRNA-like small RNA interacts with and modulates p-ERM proteins in human somatic cells. Nature communications, 6(1), 1-12.
piRNA specific characteristics:
- length in between 24-35 nucleotides
expression data:
found in lung bronchial epithelial cells

## Slide 8
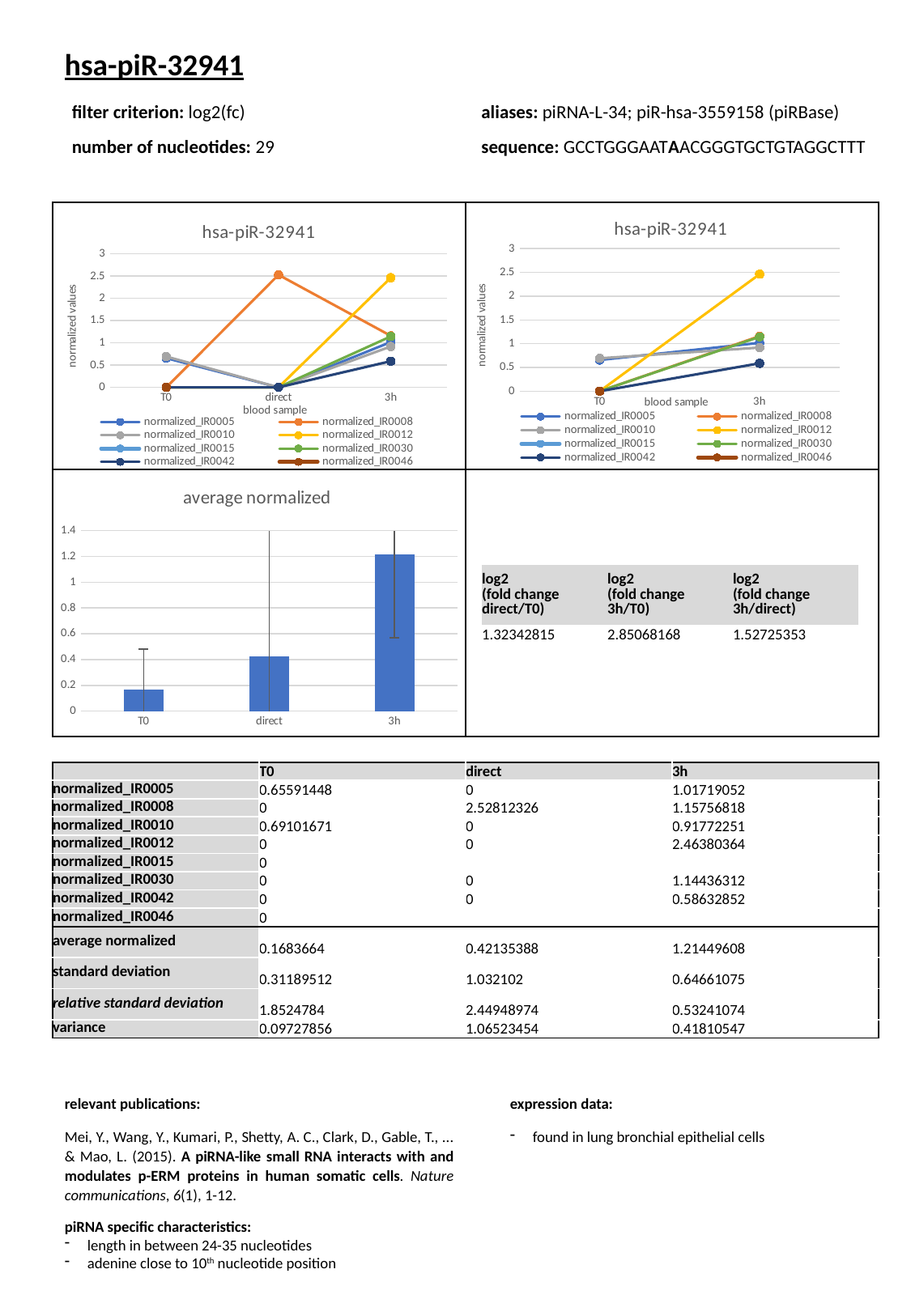

hsa-piR-32941
| filter criterion: log2(fc) | aliases: piRNA-L-34; piR-hsa-3559158 (piRBase) |
| --- | --- |
| number of nucleotides: 29 | sequence: GCCTGGGAATAACGGGTGCTGTAGGCTTT |
[unsupported chart]
[unsupported chart]
### Chart: average normalized
| Category | Durchschnitt_normalized |
|---|---|
| T0 | 0.16836639910847379 |
| direct | 0.4213538774247862 |
| 3h | 1.214496082612923 || log2 (fold change direct/T0) | log2 (fold change 3h/T0) | log2 (fold change 3h/direct) |
| --- | --- | --- |
| 1.32342815 | 2.85068168 | 1.52725353 |
| | T0 | direct | 3h |
| --- | --- | --- | --- |
| normalized\_IR0005 | 0.65591448 | 0 | 1.01719052 |
| normalized\_IR0008 | 0 | 2.52812326 | 1.15756818 |
| normalized\_IR0010 | 0.69101671 | 0 | 0.91772251 |
| normalized\_IR0012 | 0 | 0 | 2.46380364 |
| normalized\_IR0015 | 0 | | |
| normalized\_IR0030 | 0 | 0 | 1.14436312 |
| normalized\_IR0042 | 0 | 0 | 0.58632852 |
| normalized\_IR0046 | 0 | | |
| average normalized | 0.1683664 | 0.42135388 | 1.21449608 |
| standard deviation | 0.31189512 | 1.032102 | 0.64661075 |
| relative standard deviation | 1.8524784 | 2.44948974 | 0.53241074 |
| variance | 0.09727856 | 1.06523454 | 0.41810547 |
relevant publications:
Mei, Y., Wang, Y., Kumari, P., Shetty, A. C., Clark, D., Gable, T., ... & Mao, L. (2015). A piRNA-like small RNA interacts with and modulates p-ERM proteins in human somatic cells. Nature communications, 6(1), 1-12.
piRNA specific characteristics:
length in between 24-35 nucleotides
adenine close to 10th nucleotide position
expression data:
found in lung bronchial epithelial cells

## Slide 9
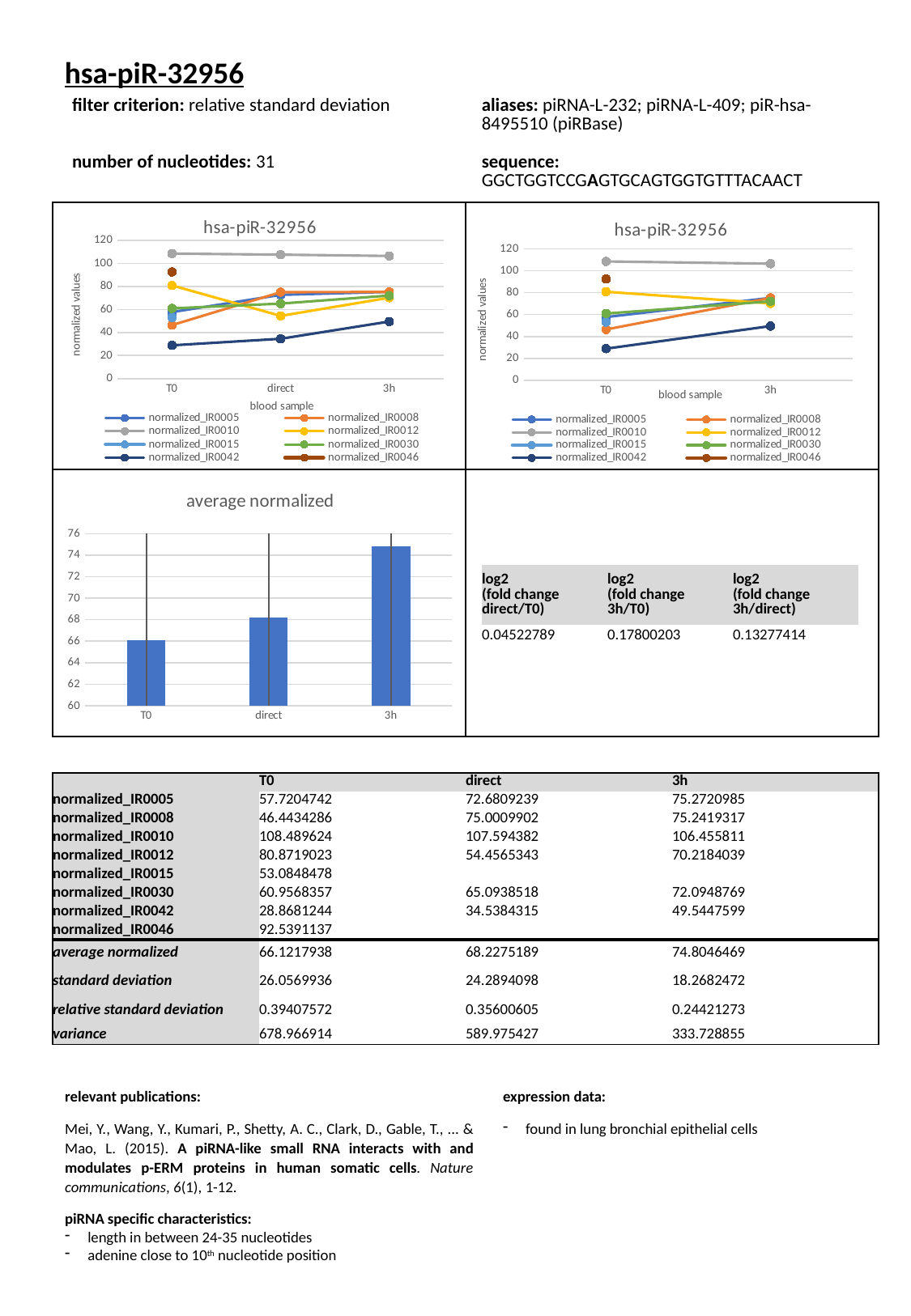

hsa-piR-32956
| filter criterion: relative standard deviation | aliases: piRNA-L-232; piRNA-L-409; piR-hsa-8495510 (piRBase) |
| --- | --- |
| number of nucleotides: 31 | sequence: GGCTGGTCCGAGTGCAGTGGTGTTTACAACT |
[unsupported chart]
[unsupported chart]
### Chart: average normalized
| Category | Durchschnitt_normalized |
|---|---|
| T0 | 66.12179384728688 |
| direct | 68.22751894524892 |
| 3h | 74.80464692814363 || log2 (fold change direct/T0) | log2 (fold change 3h/T0) | log2 (fold change 3h/direct) |
| --- | --- | --- |
| 0.04522789 | 0.17800203 | 0.13277414 |
| | T0 | direct | 3h |
| --- | --- | --- | --- |
| normalized\_IR0005 | 57.7204742 | 72.6809239 | 75.2720985 |
| normalized\_IR0008 | 46.4434286 | 75.0009902 | 75.2419317 |
| normalized\_IR0010 | 108.489624 | 107.594382 | 106.455811 |
| normalized\_IR0012 | 80.8719023 | 54.4565343 | 70.2184039 |
| normalized\_IR0015 | 53.0848478 | | |
| normalized\_IR0030 | 60.9568357 | 65.0938518 | 72.0948769 |
| normalized\_IR0042 | 28.8681244 | 34.5384315 | 49.5447599 |
| normalized\_IR0046 | 92.5391137 | | |
| average normalized | 66.1217938 | 68.2275189 | 74.8046469 |
| standard deviation | 26.0569936 | 24.2894098 | 18.2682472 |
| relative standard deviation | 0.39407572 | 0.35600605 | 0.24421273 |
| variance | 678.966914 | 589.975427 | 333.728855 |
relevant publications:
Mei, Y., Wang, Y., Kumari, P., Shetty, A. C., Clark, D., Gable, T., ... & Mao, L. (2015). A piRNA-like small RNA interacts with and modulates p-ERM proteins in human somatic cells. Nature communications, 6(1), 1-12.
piRNA specific characteristics:
length in between 24-35 nucleotides
adenine close to 10th nucleotide position
expression data:
found in lung bronchial epithelial cells

## Slide 10
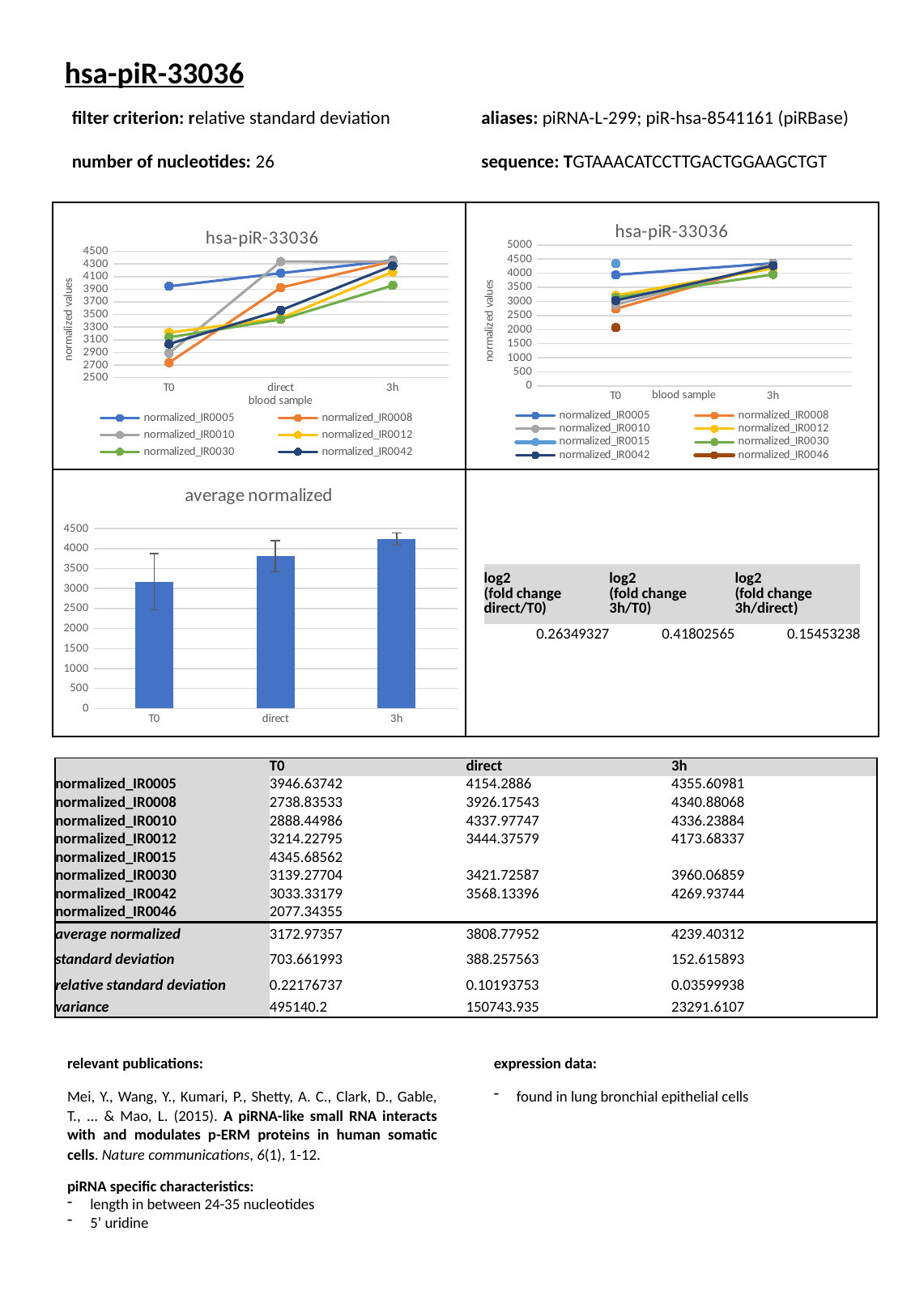

hsa-piR-33036
| filter criterion: relative standard deviation | aliases: piRNA-L-299; piR-hsa-8541161 (piRBase) |
| --- | --- |
| number of nucleotides: 26 | sequence: TGTAAACATCCTTGACTGGAAGCTGT |
[unsupported chart]
### Chart:
| Category | normalized_IR0005 | normalized_IR0008 | normalized_IR0010 | normalized_IR0012 | normalized_IR0030 | normalized_IR0042 |
|---|---|---|---|---|---|---|
| T0 | 3946.637421626419 | 2738.835331506559 | 2888.449862936835 | 3214.22794864118 | 3139.277039488212 | 3033.33179006882 |
| direct | 4154.288595291959 | 3926.175429844158 | 4337.977467883789 | 3444.375791976039 | 3421.72587136718 | 3568.133963900432 |
| 3h | 4355.609805716611 | 4340.880677871927 | 4336.238842788628 | 4173.683374129816 | 3960.068593125696 | 4269.937441678635 |
### Chart: average normalized
| Category | Durchschnitt_normalized |
|---|---|
| T0 | 3172.9735709024676 |
| direct | 3808.7795200439264 |
| 3h | 4239.403122551886 || log2 (fold change direct/T0) | log2 (fold change 3h/T0) | log2 (fold change 3h/direct) |
| --- | --- | --- |
| 0.26349327 | 0.41802565 | 0.15453238 |
| | T0 | direct | 3h |
| --- | --- | --- | --- |
| normalized\_IR0005 | 3946.63742 | 4154.2886 | 4355.60981 |
| normalized\_IR0008 | 2738.83533 | 3926.17543 | 4340.88068 |
| normalized\_IR0010 | 2888.44986 | 4337.97747 | 4336.23884 |
| normalized\_IR0012 | 3214.22795 | 3444.37579 | 4173.68337 |
| normalized\_IR0015 | 4345.68562 | | |
| normalized\_IR0030 | 3139.27704 | 3421.72587 | 3960.06859 |
| normalized\_IR0042 | 3033.33179 | 3568.13396 | 4269.93744 |
| normalized\_IR0046 | 2077.34355 | | |
| average normalized | 3172.97357 | 3808.77952 | 4239.40312 |
| standard deviation | 703.661993 | 388.257563 | 152.615893 |
| relative standard deviation | 0.22176737 | 0.10193753 | 0.03599938 |
| variance | 495140.2 | 150743.935 | 23291.6107 |
relevant publications:
Mei, Y., Wang, Y., Kumari, P., Shetty, A. C., Clark, D., Gable, T., ... & Mao, L. (2015). A piRNA-like small RNA interacts with and modulates p-ERM proteins in human somatic cells. Nature communications, 6(1), 1-12.
piRNA specific characteristics:
length in between 24-35 nucleotides
5’ uridine
expression data:
found in lung bronchial epithelial cells

## Slide 11
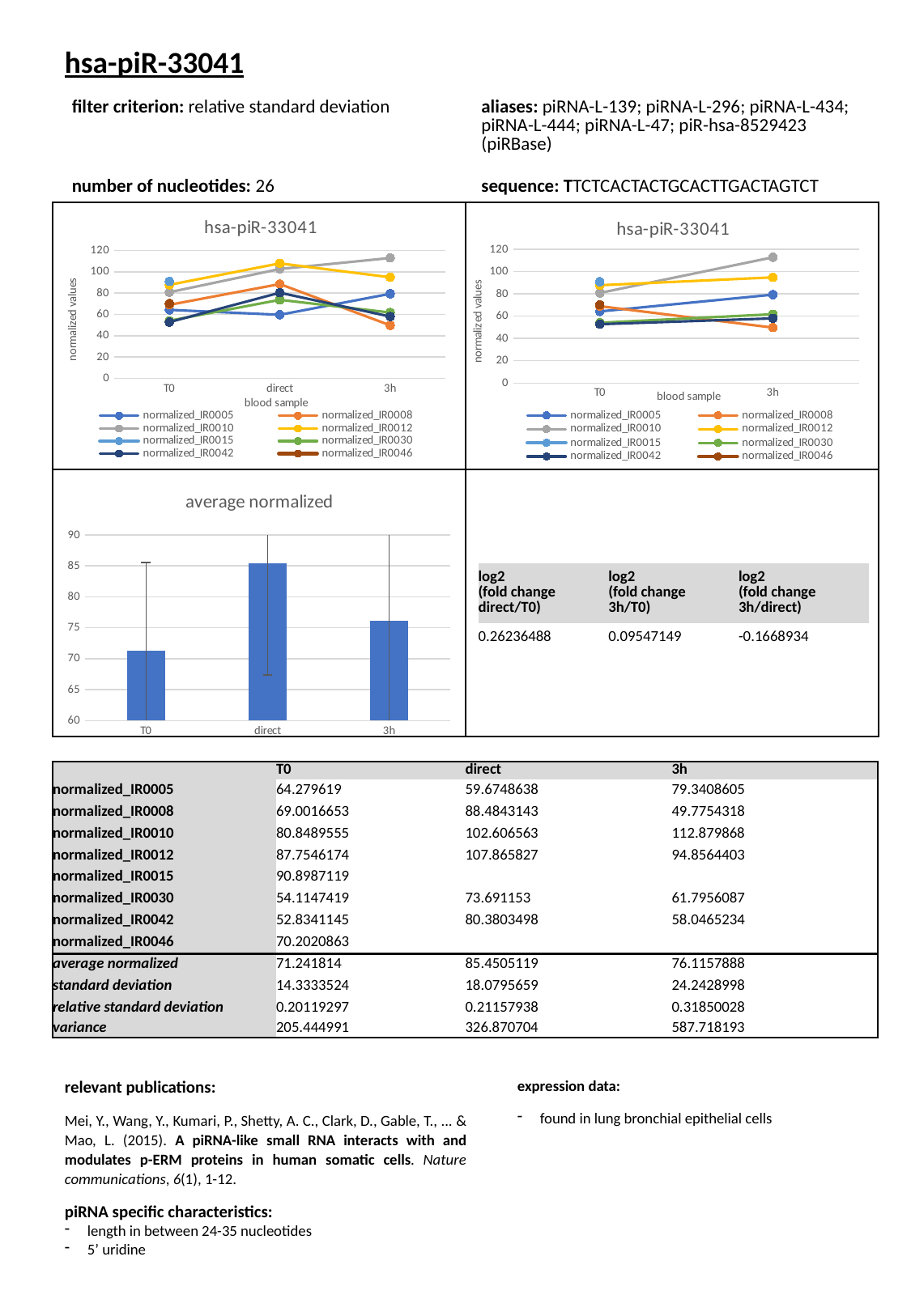

hsa-piR-33041
| filter criterion: relative standard deviation | aliases: piRNA-L-139; piRNA-L-296; piRNA-L-434; piRNA-L-444; piRNA-L-47; piR-hsa-8529423 (piRBase) |
| --- | --- |
| number of nucleotides: 26 | sequence: TTCTCACTACTGCACTTGACTAGTCT |
[unsupported chart]
[unsupported chart]
### Chart: average normalized
| Category | Durchschnitt_normalized |
|---|---|
| T0 | 71.24181398098438 |
| direct | 85.45051188082594 |
| 3h | 76.11578884501837 || log2 (fold change direct/T0) | log2 (fold change 3h/T0) | log2 (fold change 3h/direct) |
| --- | --- | --- |
| 0.26236488 | 0.09547149 | -0.1668934 |
| | T0 | direct | 3h |
| --- | --- | --- | --- |
| normalized\_IR0005 | 64.279619 | 59.6748638 | 79.3408605 |
| normalized\_IR0008 | 69.0016653 | 88.4843143 | 49.7754318 |
| normalized\_IR0010 | 80.8489555 | 102.606563 | 112.879868 |
| normalized\_IR0012 | 87.7546174 | 107.865827 | 94.8564403 |
| normalized\_IR0015 | 90.8987119 | | |
| normalized\_IR0030 | 54.1147419 | 73.691153 | 61.7956087 |
| normalized\_IR0042 | 52.8341145 | 80.3803498 | 58.0465234 |
| normalized\_IR0046 | 70.2020863 | | |
| average normalized | 71.241814 | 85.4505119 | 76.1157888 |
| standard deviation | 14.3333524 | 18.0795659 | 24.2428998 |
| relative standard deviation | 0.20119297 | 0.21157938 | 0.31850028 |
| variance | 205.444991 | 326.870704 | 587.718193 |
relevant publications:
Mei, Y., Wang, Y., Kumari, P., Shetty, A. C., Clark, D., Gable, T., ... & Mao, L. (2015). A piRNA-like small RNA interacts with and modulates p-ERM proteins in human somatic cells. Nature communications, 6(1), 1-12.
piRNA specific characteristics:
length in between 24-35 nucleotides
5’ uridine
expression data:
found in lung bronchial epithelial cells

## Slide 12
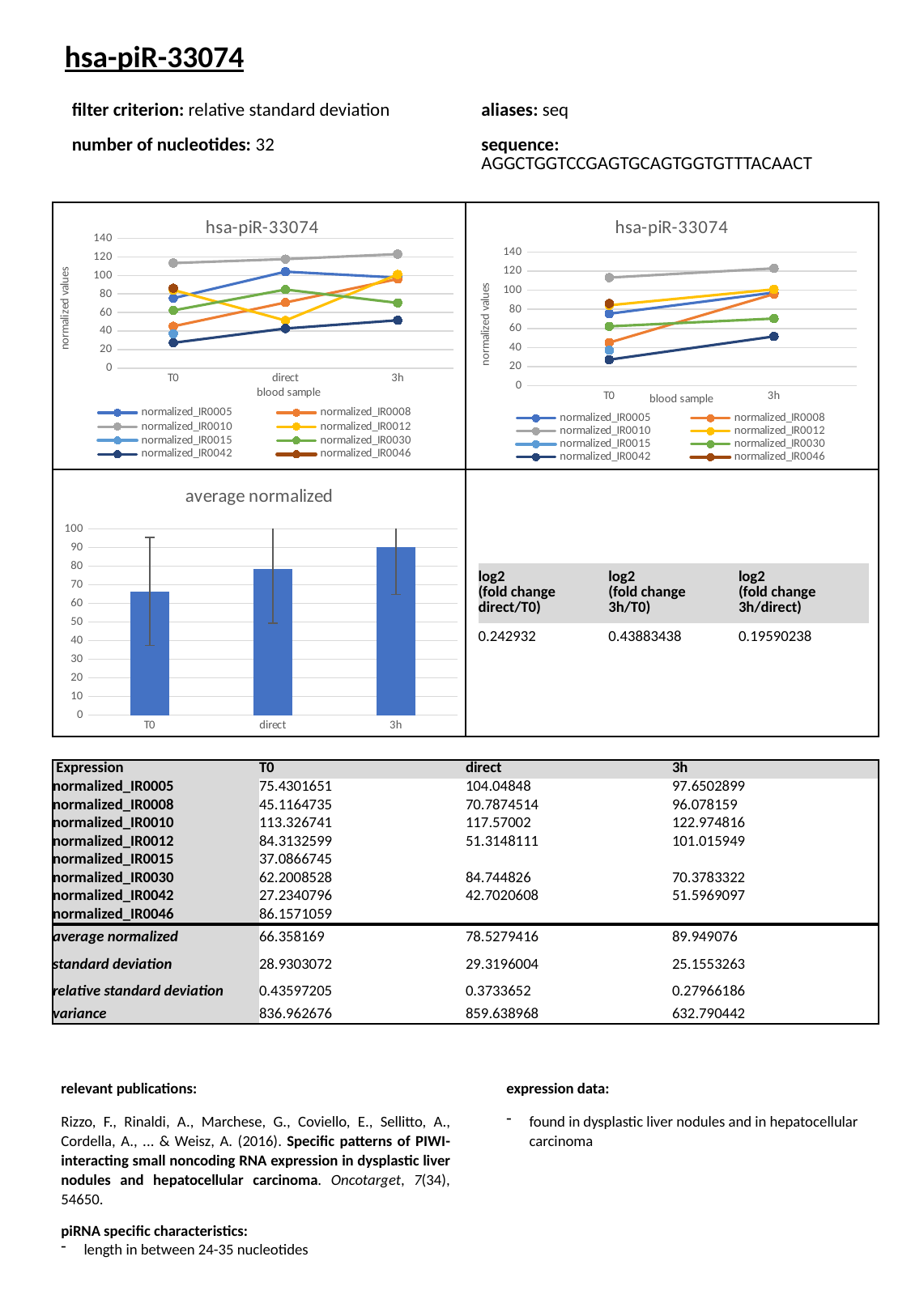

hsa-piR-33074
| filter criterion: relative standard deviation | aliases: seq |
| --- | --- |
| number of nucleotides: 32 | sequence: AGGCTGGTCCGAGTGCAGTGGTGTTTACAACT |
[unsupported chart]
[unsupported chart]
### Chart: average normalized
| Category | Durchschnitt_normalized |
|---|---|
| T0 | 66.35816903647233 |
| direct | 78.52794164253089 |
| 3h | 89.94907601041565 || log2 (fold change direct/T0) | log2 (fold change 3h/T0) | log2 (fold change 3h/direct) |
| --- | --- | --- |
| 0.242932 | 0.43883438 | 0.19590238 |
| Expression | T0 | direct | 3h |
| --- | --- | --- | --- |
| normalized\_IR0005 | 75.4301651 | 104.04848 | 97.6502899 |
| normalized\_IR0008 | 45.1164735 | 70.7874514 | 96.078159 |
| normalized\_IR0010 | 113.326741 | 117.57002 | 122.974816 |
| normalized\_IR0012 | 84.3132599 | 51.3148111 | 101.015949 |
| normalized\_IR0015 | 37.0866745 | | |
| normalized\_IR0030 | 62.2008528 | 84.744826 | 70.3783322 |
| normalized\_IR0042 | 27.2340796 | 42.7020608 | 51.5969097 |
| normalized\_IR0046 | 86.1571059 | | |
| average normalized | 66.358169 | 78.5279416 | 89.949076 |
| standard deviation | 28.9303072 | 29.3196004 | 25.1553263 |
| relative standard deviation | 0.43597205 | 0.3733652 | 0.27966186 |
| variance | 836.962676 | 859.638968 | 632.790442 |
relevant publications:
Rizzo, F., Rinaldi, A., Marchese, G., Coviello, E., Sellitto, A., Cordella, A., ... & Weisz, A. (2016). Specific patterns of PIWI-interacting small noncoding RNA expression in dysplastic liver nodules and hepatocellular carcinoma. Oncotarget, 7(34), 54650.
piRNA specific characteristics:
length in between 24-35 nucleotides
expression data:
found in dysplastic liver nodules and in hepatocellular carcinoma

## Slide 13
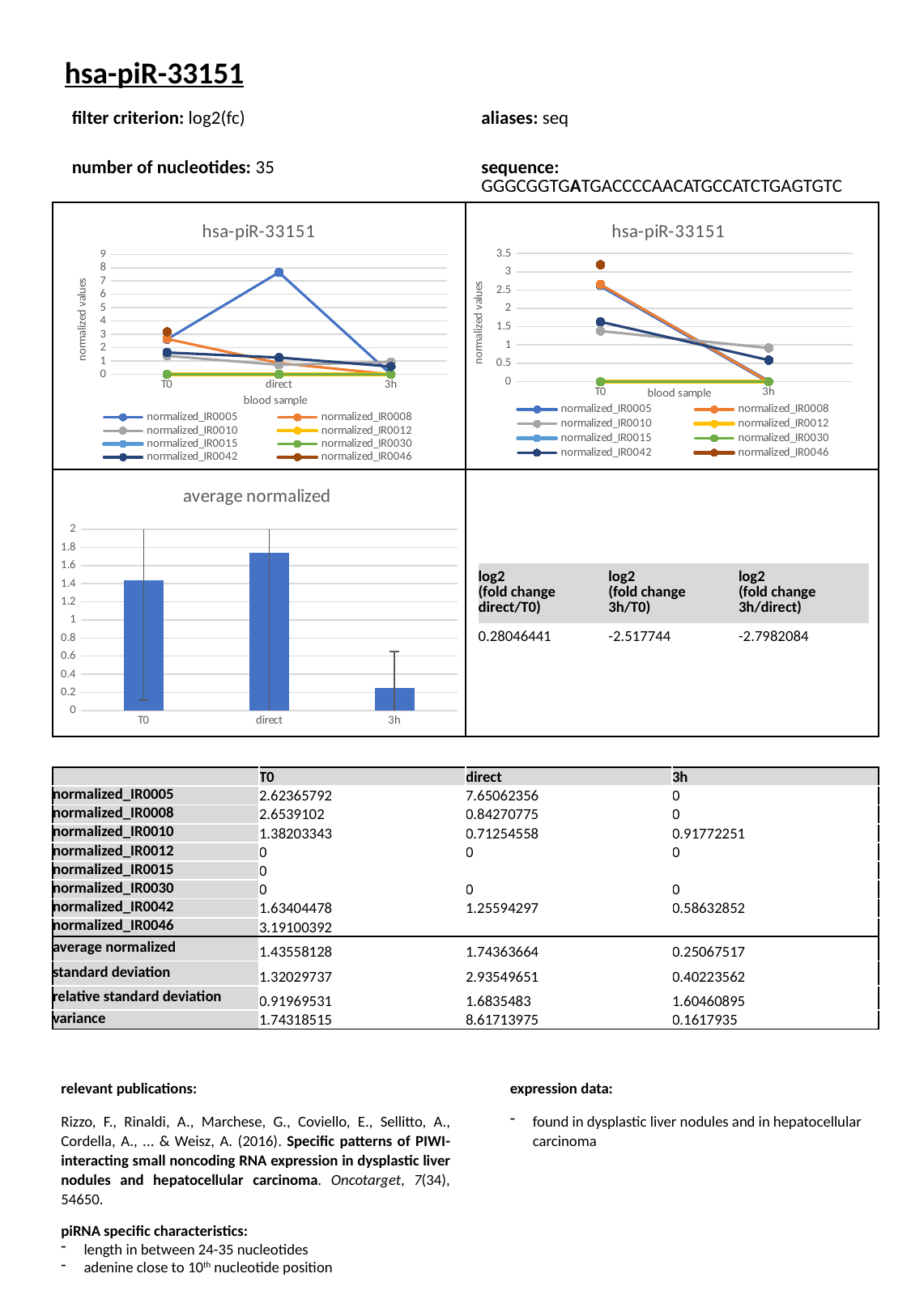

hsa-piR-33151
| filter criterion: log2(fc) | aliases: seq |
| --- | --- |
| number of nucleotides: 35 | sequence: GGGCGGTGATGACCCCAACATGCCATCTGAGTGTC |
[unsupported chart]
[unsupported chart]
### Chart: average normalized
| Category | Durchschnitt_normalized |
|---|---|
| T0 | 1.4355812811493043 |
| direct | 1.7436366433729413 |
| 3h | 0.2506751709488862 || log2 (fold change direct/T0) | log2 (fold change 3h/T0) | log2 (fold change 3h/direct) |
| --- | --- | --- |
| 0.28046441 | -2.517744 | -2.7982084 |
| | T0 | direct | 3h |
| --- | --- | --- | --- |
| normalized\_IR0005 | 2.62365792 | 7.65062356 | 0 |
| normalized\_IR0008 | 2.6539102 | 0.84270775 | 0 |
| normalized\_IR0010 | 1.38203343 | 0.71254558 | 0.91772251 |
| normalized\_IR0012 | 0 | 0 | 0 |
| normalized\_IR0015 | 0 | | |
| normalized\_IR0030 | 0 | 0 | 0 |
| normalized\_IR0042 | 1.63404478 | 1.25594297 | 0.58632852 |
| normalized\_IR0046 | 3.19100392 | | |
| average normalized | 1.43558128 | 1.74363664 | 0.25067517 |
| standard deviation | 1.32029737 | 2.93549651 | 0.40223562 |
| relative standard deviation | 0.91969531 | 1.6835483 | 1.60460895 |
| variance | 1.74318515 | 8.61713975 | 0.1617935 |
relevant publications:
Rizzo, F., Rinaldi, A., Marchese, G., Coviello, E., Sellitto, A., Cordella, A., ... & Weisz, A. (2016). Specific patterns of PIWI-interacting small noncoding RNA expression in dysplastic liver nodules and hepatocellular carcinoma. Oncotarget, 7(34), 54650.
piRNA specific characteristics:
length in between 24-35 nucleotides
adenine close to 10th nucleotide position
expression data:
found in dysplastic liver nodules and in hepatocellular carcinoma
