# Supplementary figures and images for: Patterns of Circulating piRNAs in the Context of a Single Bout of Exercise: Potential Biomarkers of Exercise-Induced Adaptation?
Source: Noncoding RNA. 2025 Jun 16;11(3):46. doi: 10.3390/ncrna11030046 (PMC12195705; doi:10.3390/ncrna11030046)

## Slide 1
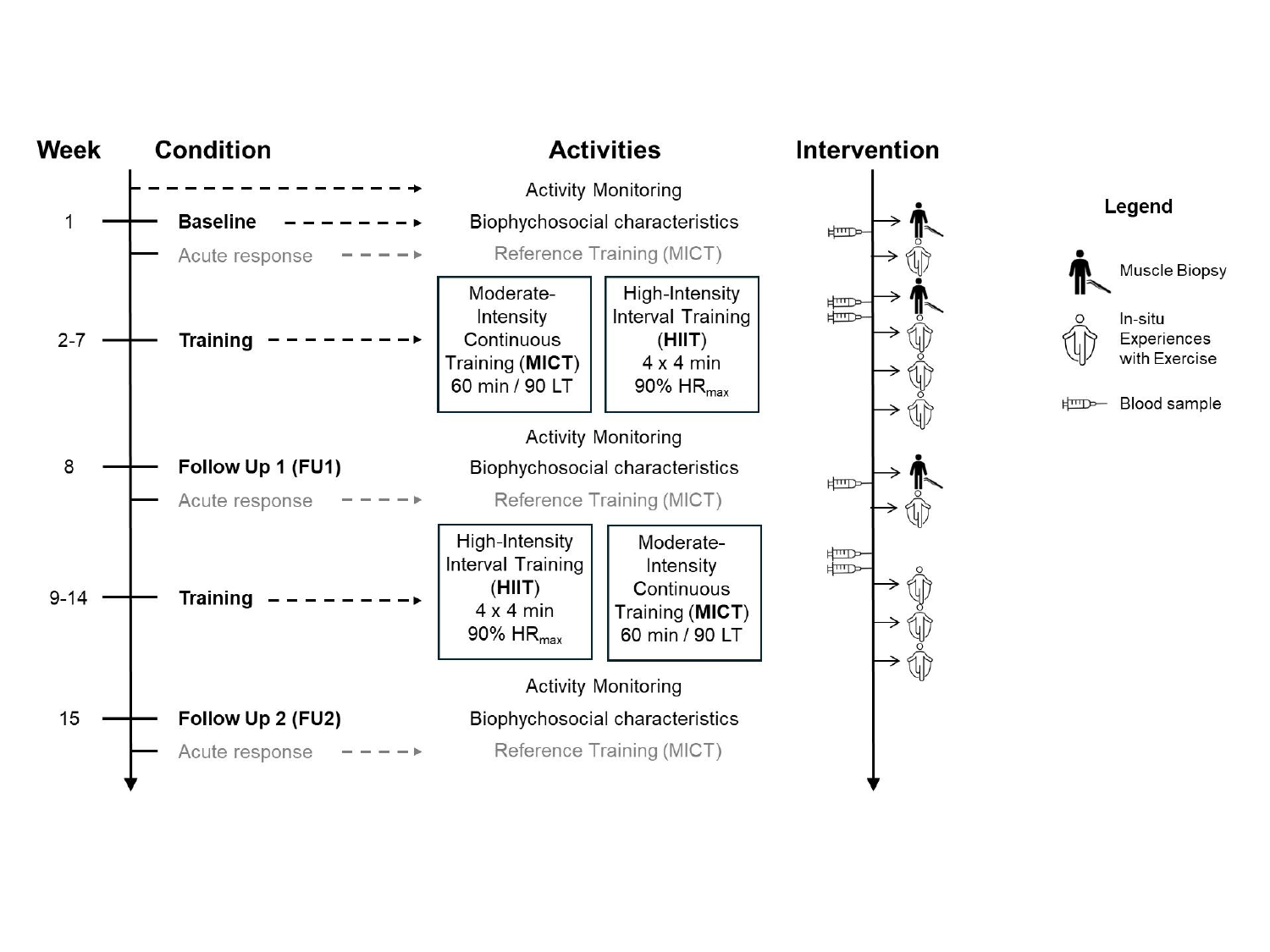

Supplement: Supplementary file 1 [file ncrna-11-00046-s001.zip › Figure S1 revised.pptx]
